# Adaptation delay causes a burst of mutations in bacteria responding to oxidative stress

**DOI:** 10.15252/embr.202255640

**Published:** 2022-11-17

**Authors:** Valentine Lagage, Victor Chen, Stephan Uphoff

**Affiliations:** ^1^ Department of Biochemistry University of Oxford Oxford UK

**Keywords:** DNA damage, mutation, oxidative stress, single‐molecule imaging, stress response, DNA Replication, Recombination & Repair, Genetics, Gene Therapy & Genetic Disease, Microbiology, Virology & Host Pathogen Interaction

## Abstract

Understanding the interplay between phenotypic and genetic adaptation is a focus of evolutionary biology. In bacteria, the oxidative stress response prevents mutagenesis by reactive oxygen species (ROS). We hypothesise that the stress response dynamics can therefore affect the timing of the mutation supply that fuels genetic adaptation to oxidative stress. We uncover that sudden hydrogen peroxide stress causes a burst of mutations. By developing single‐molecule and single‐cell microscopy methods, we determine how these mutation dynamics arise from phenotypic adaptation mechanisms. H_2_O_2_ signalling by the transcription factor OxyR rapidly induces ROS‐scavenging enzymes. However, an adaptation delay leaves cells vulnerable to the mutagenic and toxic effects of hydroxyl radicals generated by the Fenton reaction. Resulting DNA damage is counteracted by a spike in DNA repair activities during the adaptation delay. Absence of a mutation burst in cells with prior stress exposure or constitutive OxyR activation shows that the timing of phenotypic adaptation directly controls stress‐induced mutagenesis. Similar observations for alkylation stress show that mutation bursts are a general phenomenon associated with adaptation delays.

## Graphical abstract

*Escherichia coli* cells experience a burst of DNA replication errors upon H_2_O_2_ treatment. This burst is fuelled by the iron‐catalysed Fenton reaction and terminated when the OxyR‐dependent stress response induces H_2_O_2_ scavenging enzymes.

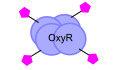

## Introduction

Phenotypic and genetic plasticity are at the roots of the remarkable capacity of bacteria for adaptation. Stress responses induce phenotypic changes that protect bacteria against adverse conditions in the environment, thus opening a window of opportunity during which adaptive mutations may arise that provide long‐term heritable stress resistance. The relative timing of phenotypic and genetic changes is critical to the process of adaptation. On the one hand, rapid phenotypic responses allow cells to survive transient stresses (Storz & Hengge‐Aronis, 2010) without having to acquire permanent genetic changes, which are rarely beneficial to the individual when the stress has passed (Robert *et al*, 2018). On the other hand, severe stress conditions can quickly drive a bacterial population to extinction. In such situations, protection from phenotypic responses is insufficient and population survival relies on evolutionary rescue via the rapid emergence of adaptive mutations (Bell, 2017). These mutations can be present as pre‐existing genetic variation in a population due to spontaneous mutagenesis prior to stress exposure. However, cell stress also induces *de‐novo* mutagenesis (Bjelland & Seeberg, 2003; Galhardo *et al*, 2007; Kohanski *et al*, 2010a; Uphoff, 2018; Wang *et al*, 2018), which offers the potential evolutionary benefit of confining genetic changes to times when they are most likely to be beneficial. Whether stress‐induced mutagenesis plays a significant role in adaptation is an open question that depends on its magnitude relative to the spontaneous mutation rate and its timing relative to the population dynamics, both of which remain uncertain. Furthermore, even well‐defined treatments of bacteria with a single stress agent usually activate a complex network of stress responses involving genes that counteract mutagenesis and genes that actively induce mutations (Kreuzer, 2013; Uphoff, 2018; Pribis *et al*, 2019), making it hard to predict how the stress itself and the cellular responses together affect mutation rates. To date, experimental evidence for the actual temporal order and inter‐dependence of phenotypic and genetic changes during stress adaptation is lacking because conventional methods for detecting mutations do not resolve phenotypic responses and vice versa. In this study, we asked how mutation rates change when bacteria experience stress, and how these dynamics relate to the underlying molecular mechanisms of adaptation. It has now become possible to address these questions owing to a new live‐cell microscopy approach to measure DNA replication error rates while simultaneously monitoring cell growth, morphology and gene expression dynamics (Robert *et al*, 2018; Uphoff, 2018).

Amongst the most common and harmful stress factors that threaten bacteria are reactive oxygen species (ROS), which are constantly formed inside cells as a by‐product of their metabolism (Imlay, 2008). ROS levels increase under various environmental conditions. For example, sudden bursts of ROS are generated by immune cells to fight bacterial infections (Fang *et al*, 2016), when bacteria are exposed to radiation (Imlay, 2015) and possibly bactericidal antibiotics (Kohanski *et al*, 2010a; Giroux *et al*, 2017; Hong *et al*, 2019), and during competition with other bacterial species (Dong *et al*, 2015). Hydrogen peroxide (H_2_O_2_) is one of the major ROS and can cause damage to proteins, lipids and DNA through the formation of hydroxyl radicals (HO^·^) via the Fenton reaction with intracellular iron (Imlay, 2008). Purine nucleobases are particularly prone to oxidation, resulting in premutagenic lesions, which can form mismatched base pairs during DNA replication, ultimately leading to mutations if not repaired (Bjelland & Seeberg, 2003). To counter this omnipotent threat to genome stability, dedicated enzymes of the DNA Base Excision Repair (BER) pathway revert premutagenic oxidative lesions before they turn into mutations (Friedberg, 2005). As a primary defence against ROS toxicity, many bacteria rely on catalase and peroxiredoxin enzymes to scavenge intracellular H_2_O_2_ (Imlay, 2008). Although the specific mechanisms vary between bacterial species, the expression of ROS‐scavenging enzymes is generally regulated via redox‐sensitive transcription factors such as OxyR, which control oxidative stress responses that substantially increase ROS tolerance (Storz *et al*, 1990).

Over time, prolonged oxidative stress leads to the selection of adaptive mutations in a variety of targets that affect iron homeostasis and cell motility (Rodriguez‐Rojas *et al*, 2020), increase the expression of catalase (Li *et al*, 2014) or lead to constitutive OxyR induction (Gundlach & Winter, 2014; Anand *et al*, 2020). The mutagenic effects of ROS are thought to accelerate the evolution of antibiotic resistance and host adaptation (Weitzman & Stossel, 1981; Kohanski *et al*, 2010a; Long *et al*, 2016; Wang *et al*, 2018). Interestingly, the mutation supply driving such genetic adaptation was shown to depend on the temporal pattern of ROS exposure (Rodriguez‐Rojas *et al*, 2020), suggesting that mutation rates may be dynamically modulated by changes in the stress level and the cellular stress responses.

## Results

### Monitoring adaptation of *E. coli* to H_2_O_2_ treatment in microfluidic chips

**Figure 1:**
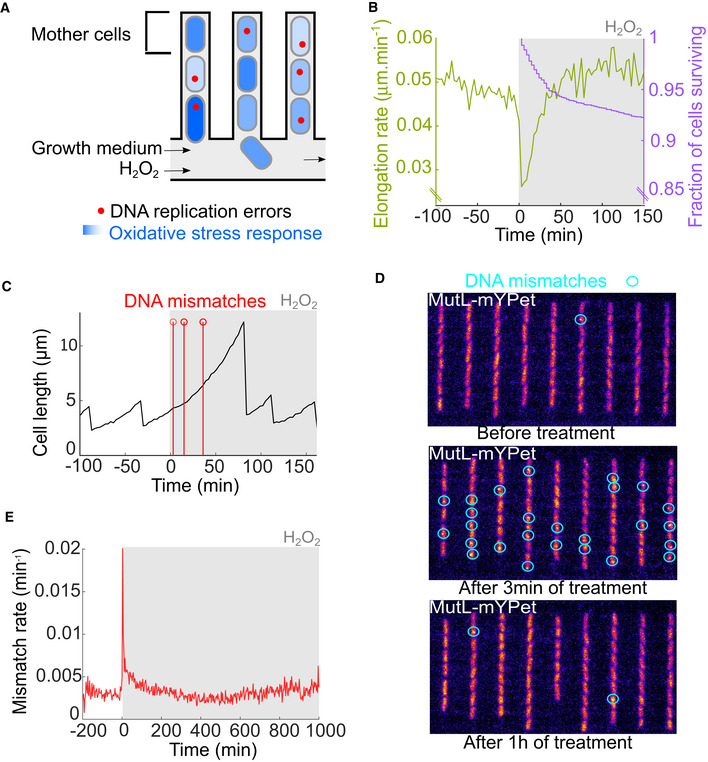
H_2_O_2_ treatment causes a burst of mutation Single‐cell microscopy monitors DNA replication errors and fluorescent gene expression reporters for the oxidative stress response during constant H_2_O_2_ treatment using microfluidics. The “mother cells” are located at the closed end of each growth channel and can be observed continuously over multiple generations.Cell elongation rate (green) and fraction of cells surviving (purple) in the microfluidic chip during constant treatment with 100 μM H_2_O_2_ added at time 0 (6,657 cells, eight biological replicates).Example time trace of one mother cell growing and dividing before and during constant treatment with 100 μM H_2_O_2_ added at time 0. Red markers indicate the timing of DNA mismatch events.Snapshots of MutL‐mYPet foci (blue circles) marking DNA mismatches in cells before treatment, and after 3 min and 1 h of constant treatment with 100 μM H_2_O_2_.Rate of DNA mismatches per cell per minute before and during constant treatment with 100 μM H_2_O_2_ (6,655 cells, eight biological replicates).

**Figure EV1:**
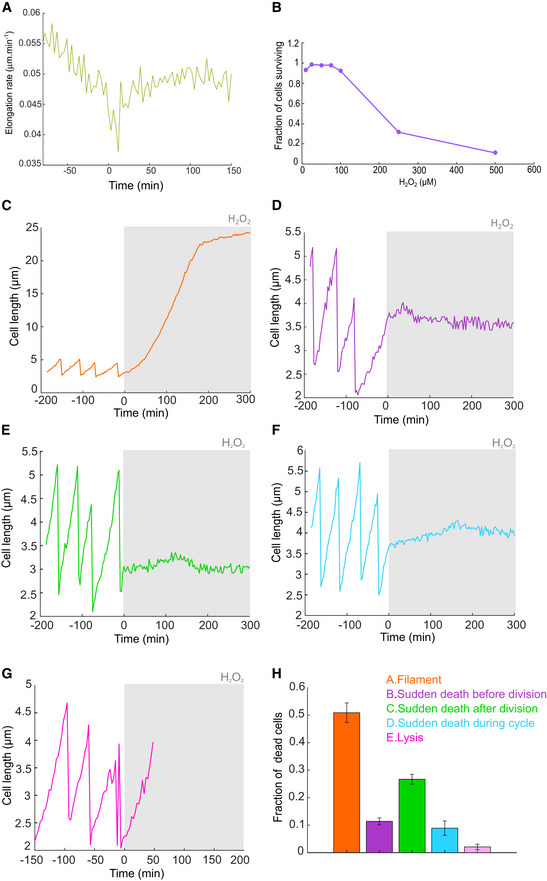
Cell elongation rate with 25 μM H_2_O_2_, survival after 100 min in cells treated with different doses of H_2_O_2_ and cell length traces showing different types of cell death with 100 μM H_2_O_2_ treatment Cell elongation rate with 25 μM H_2_O_2_ (1,830 cells, three biological replicates).Fraction of cells surviving after 100 min of treatment with different doses of H_2_O_2_ (10 μM (656 cells, one biological replicate), 25 μM (1,314 cells, two biological replicates), 50 μM (476 cells, one biological replicate), 75 μM (471 cells, one biological replicate), 100 μM (6,657 cells, eight biological replicates), 250 μM (335 cells, one biological replicate), 500 μM (772 cells, one biological replicate).Example cell length trace showing filamentation.Example cell length trace showing growth arrest just before cell division.Example cell length trace showing growth arrest just after cell division.Example cell length trace showing growth arrest during the cell cycle.Example cell length trace showing lysis.Percentage of cells for each type of cell death ((6,562 cells, eight biological replicates), mean ± SEM).

We set out to monitor the timing of phenotypic adaptation and mutagenesis of *E. coli* cells exposed to H_2_O_2_ via live‐cell microscopy. Conventional bulk culture assays typically employ a single H_2_O_2_ bolus, which decays in the medium over time due to efficient ROS scavenging by the bacteria. Because of this, induction of prolonged oxidative stress had typically required treatment with very high (~millimolar) H_2_O_2_ concentrations, which are outside the range experienced by bacteria in nature (Li & Imlay, 2018). To avoid this and to uncouple the dynamics of the bacterial responses from changes in the H_2_O_2_ concentrations in the media, we imaged cells growing inside the “mother machine” microfluidic device (Wang *et al*, 2010; Uphoff, 2018). Individual cells are maintained within μm‐sized channels under exponential growth conditions over tens of generations and supplied with a continuous inflow of fresh media and H_2_O_2_ treatment at a constant concentration (Fig 1A). The individual cells at the closed end of each growth channel (named “mother cells”) can be monitored continuously, while their daughter cells are pushed towards the open ends of the channels and washed out with the media outflow. We used time‐lapse epifluorescence microscopy to measure cell elongation rates (change in cell length) and generation times (interval between consecutive cell divisions) via automated segmentation of the cell shape aided by a cytoplasmic mKate2 fluorescent protein. An experiment typically monitored 500–1,000 individual mother cells, each in a separate growth channel. After a period of unperturbed growth for several generations, we switched the media inflow to expose cells to a constant concentration of H_2_O_2_ that is continuously replenished by the fluidics system. H_2_O_2_ crosses the *E. coli* cell envelope readily (Seaver & Imlay, 2001b), and the elongation rate dropped two‐fold immediately after the start of 100 μM H_2_O_2_ treatment (Fig 1B). We observed a partial inhibition of cell elongation for H_2_O_2_ concentrations as low as 25 μM, demonstrating that physiologically‐relevant H_2_O_2_ concentrations are sufficient to cause detrimental oxidative stress (Fig EV1A). Greater than 90% of cells survived treatments up to 100 μM H_2_O_2_, but cell survival dropped steeply for higher concentrations (Fig EV1B). For surviving cells, elongation rates (Fig 1C) and generation times (Appendix Fig S1) recovered to pretreatment rates after an adaptation lag period. The adaptation lag was also marked by a transient period of increased cell mortality, which manifested either as the irreversible cessation of elongation, sudden cell lysis or extreme filamentation that caused the escape of the cell from the growth channel (Fig EV1C–H). Due to the constant treatment, the recovery of cell elongation and viability cannot be attributed to decay of H_2_O_2_ in the medium but rather reflect the phenotypic adaptation that enables unperturbed growth in the presence of H_2_O_2_. However, an initial lag period before adaptation leaves the bacteria vulnerable to the toxic effects of H_2_O_2_, which have been attributed to the oxidation of DNA (Imlay & Linn, 1986) and proteins (Ezraty *et al*, 2017) and a shutdown of protein synthesis (Zhong *et al*, 2015; Zhu & Dai, 2019).

### Sudden oxidative stress causes a burst of mutations

We next asked how the timing of phenotypic adaptation relates to the rates of genetic change during oxidative stress. To this end, we employed an imaging‐based method to monitor mutagenesis in live bacteria (Robert *et al*, 2018; Uphoff, 2018). Point mutations arise during DNA replication due to the erroneous incorporation of nucleotides that do not match the DNA template sequence. Certain chemical modifications of bases on the template strands are highly mutagenic due to their propensity to form DNA mismatches. While most DNA replication errors are corrected by the DNA mismatch repair (MMR) system, a small fraction of mismatches (1%) escapes repair and is converted into stable point mutations during the next replication cycle (Long *et al*, 2018). Fusion of the MMR enzyme MutL to the fluorescent protein mYPet yields a visual reporter for DNA replication errors in live cells, marked by the assembly of MutL‐mYPet molecules in foci at a DNA mismatch (Elez *et al*, 2010; Uphoff, 2018). Notably, the frequency of MutL‐mYPet foci reports on the rate of DNA replication errors, irrespective of whether a DNA mismatch ultimately turns into a mutation or is repaired. Nevertheless, we showed that the rate of MutL‐mYPet foci (mismatch rate) in a cell population correlates with the frequency of genomic mutations, validating its use as a mutation reporter (Vincent & Uphoff, 2021). Because DNA mismatches are rare and transient, we monitored thousands of cells in order to obtain a reliable estimate of the instantaneous mismatch rate per cell per minute. As seen before (Robert *et al*, 2018; Uphoff, 2018), the mismatch rate was stable over time in untreated cells at on average 0.0029 mismatches cell^−1^ min^−1^ (Appendix Fig S2).

**Figure EV2:**
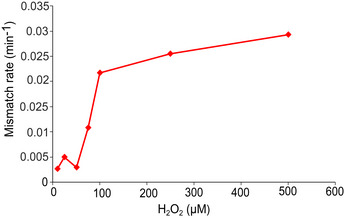
Mismatch rate peak in cells treated with different doses of H_2_O_2_ Rate of DNA mismatches per cell per minute at the peak of the mutagenesis burst for different doses of H_2_O_2_ (10 μM (651 cells, one biological replicate), 25 μM (605 cells, one biological replicate), 50 μM (one experiment, 465 cells), 75 μM (466 cells, one biological replicate), 100 μM (6,655 cells, eight biological replicates), 250 μM (331 cells, one biological replicate), 500 μM (764 cells, one biological replicate).

Treatment with 100 μM H_2_O_2_ caused a sudden ~10‐fold increase in the mismatch rate (Fig 1C). Single cells often showed multiple mismatch events shortly after the start of treatment (Fig 1D). The mismatch rate averaged over a total of 6,655 cells revealed a sharp mutagenesis burst with a duration of ~12 min and a maximum rate of 0.021 mismatches cell^−1^ min^−1^ at 4.5 min after treatment started (Fig 1E). The mutagenesis burst also occurred in the daughter cells located above the mother cell in the growth trenches (Appendix Fig S3A and B). No MutL‐mYPet foci were detected in cells with a *mutS* gene deletion either before or after H_2_O_2_ exposure (Appendix Fig S4). As MutS is required for the recognition of DNA mismatches and the recruitment of MutL, this control confirms that MutL‐mYPet foci genuinely show DNA mismatches. The amplitude of the mutagenesis burst saturated with increasing H_2_O_2_ concentration (Fig EV2), indicating that the formation of mutagenic lesions or their conversion into DNA mismatches becomes limited when cell growth is strongly inhibited by H_2_O_2_. This is consistent with the expectation that the mismatch rate scales with the replication rate (Rosche & Foster, 2000; Robert *et al*, 2018). Our observation that the mutagenesis burst also occurs at H_2_O_2_ concentrations that inhibit growth completely (> 200 μM in microfluidic culture, Fig EV2) indicates that it may contribute to the acquisition of mutations that enable evolutionary rescue during lethal stress (Rodriguez‐Rojas *et al*, 2020).

**Figure EV3:**
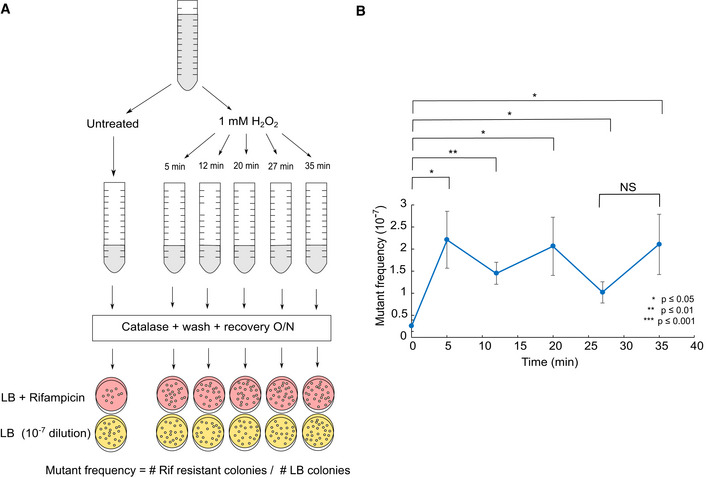
Measurement of the genomic mutation frequency confirms that H_2_O_2_ causes a mutation burst at the start of treatment Cultures were treated with 1 mM H_2_O_2_ and the treatment was stopped after 5, 12, 20, 27 and 32 min by adding catalase, then washed with M9 without H_2_O_2_ and recovered overnight before plating on LB + Rifampicin and a 10^−7^ dilution of each culture was plated on LB. Mutant frequency was quantified from the ratio of Rifampicin‐resistant colonies divided by the colony count on LB plates.The frequency of rifampicin‐resistant colonies (a reporter for mutation frequency, mean ± SEM, 6–9 experiments) increases after 5 min of 1 mM H_2_O_2_ treatment but does not increase further during prolonged treatment. Stars or NS (not significant) show the *P*‐value of the two‐sample *t*‐tests.

Reactive oxygen species cause a variety of DNA lesions (Bjelland & Seeberg, 2003). The MMR pathway does not efficiently revert all types of DNA replication errors, such as G‐A, C‐C and G‐C mispairs (Brown *et al*, 2001), and it is hence expected that these mismatches cannot be detected using the MutL‐mYPet reporter (Robert *et al*, 2018; Uphoff, 2018). To confirm the existence of the mutation burst using a different method, we measured the frequency of rifampicin‐resistant colonies, which is commonly used as a readout for genomic mutation rates (Fowler *et al*, 2003). We treated bulk cultures with 1 mM H_2_O_2_ for different lengths of time (Fig EV3A). The frequency of rifampicin‐resistant colonies increased significantly for 5 min of treatment, but there was no further increase when cultures were treated for 12 min or longer (Fig EV3B). Hence, all mutations occurred in the initial 5 min of treatment. Although these measurements lack the temporal resolution of the MutL‐mYPet reporter, the results of both methods are consistent and demonstrate that H_2_O_2_‐induced mutagenesis is confined to a brief interval after the onset of treatment.

**Figure 2:**
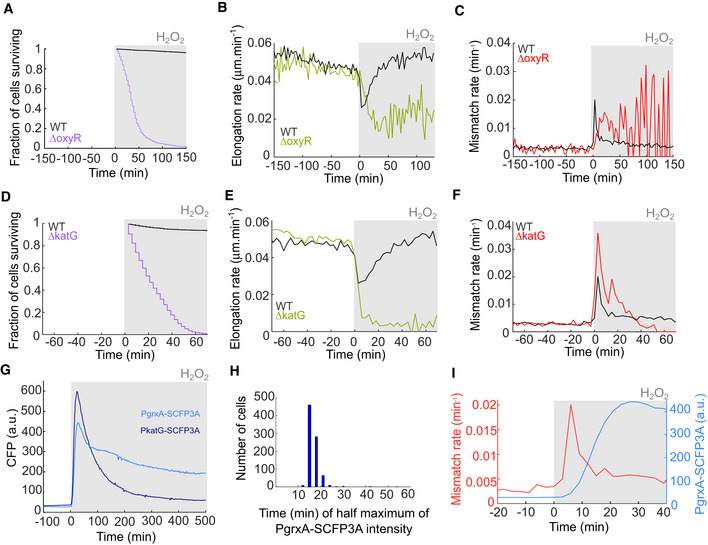
The OxyR response and induction of scavenging enzymes are required for adaptation to constant H_2_O_2_ treatment A–COxyR mutant (Δ*oxyR*) before and during constant treatment with 100 μM H_2_O_2_. (A) Fraction of cells surviving after start of treatment (734 cells, three biological replicates). (B) Cell elongation rate (668 cells, three biological replicates). (C) Rate of DNA mismatches per cell per minute (728 cells, three biological replicates).D–FCatalase deletion mutant (Δ*katG*) before and during constant treatment with 100 μM H_2_O_2_. (D) Fraction of cells surviving after start of treatment (3,040 cells, three biological replicates). (E) Cell elongation rate (2,787 cells, three biological replicates). (F) Rate of DNA mismatches per cell per minute (3,137 cells, three biological replicates).GPgrxA‐SCFP3A (1,848 cells, two biological replicates) and PkatG‐SCFP3A (988 cells, one biological replicate) fluorescence intensity before and during constant treatment with 100 μM H_2_O_2_ of WT cells.HHistogram of the lag time distribution for PgrxA‐SCFP3A to reach half‐maximal intensity after start of 100 μM H_2_O_2_ treatment (878 cells, one biological replicate).IJoined plot of the DNA mismatch rate per cell per minute (Fig 1E, 6,655 cells, eight biological replicates) and PgrxA‐SCFP3A fluorescence intensity (Fig 2G, 988 cells, one biological replicate).

### The burst of mutations coincides with a delay in the oxidative stress response

*Escherichia coli* adapts to H_2_O_2_ by induction of ROS‐scavenging enzymes, which are under the control of the transcription factor OxyR (Storz *et al*, 1990). As expected, cells with a Δ*oxyR* gene deletion were hypersensitive to H_2_O_2_ and failed to adapt to the treatment over time (Fig 2A and B). In contrast to the sharp mutagenesis burst seen in the wild‐type (WT), the lack of adaptation in the Δ*oxyR* strain led to a gradual and sustained increase of the mismatch rate in the presence of H_2_O_2_, although the observation time was limited by rapid death of Δ*oxyR* cells (Fig 2C). Furthermore, a Δ*katG* mutant lacking the OxyR‐inducible catalase, showed complete loss of growth with 100 μM H_2_O_2_ and an elevated mutagenesis burst compared with the WT (Fig 2D–F).

To address how the observed timing of phenotypic adaptation and mutagenesis relate to the gene expression dynamics of the OxyR response, we imaged transcriptional reporters for the catalase promoter PkatG fused to the fluorescent protein SCFP3A (Balleza *et al*, 2018) and the glutaredoxin 1 promoter PgrxA‐SCFP3A, expressed from low‐copy number plasmids (Zaslaver *et al*, 2006). Importantly, the time‐lapse fluorescence imaging did not induce cell toxicity (Appendix Fig S5), showing that our measurement conditions do not cause oxidative stress. As expected, both reporters were strongly induced during H_2_O_2_ treatment (Fig 2G and H), in a dose‐dependent manner (Appendix Fig S6A), and no gene induction occurred in the Δ*oxyR* strain (Appendix Fig S6B). Both reporters showed an expression peak shortly after treatment before dropping to a constant intermediate expression level. This can be explained by a high intracellular H_2_O_2_ concentration causing a strong upregulation of scavenging enzymes in naïve cells. As the scavenging capacity increases, the resulting drop in H_2_O_2_ inside cells leads to a steady‐state expression where the stress and OxyR response are balanced. The fluorescence signal of PkatG (Appendix Fig S7) and PgrxA (Fig 2H) reporters reached half‐maximal intensity after 12–15 min. This short lag time of the stress response aligns precisely with the duration of the mutagenesis burst. Indeed, simultaneous imaging of the gene expression reporters and MutL‐mYPet foci in the same cells revealed that the mutagenesis burst starts to decrease as soon as the OxyR reporter signal increases (Fig 2I). Notably, cells that acquire adaptive mutations early during the onset of stress would still rely on stress responses to bridge the phenotypic delay before the adapted phenotype is expressed (Sun *et al*, 2018).

### Single‐molecule tracking shows the dynamics of OxyR activation inside cells

**Figure 3:**
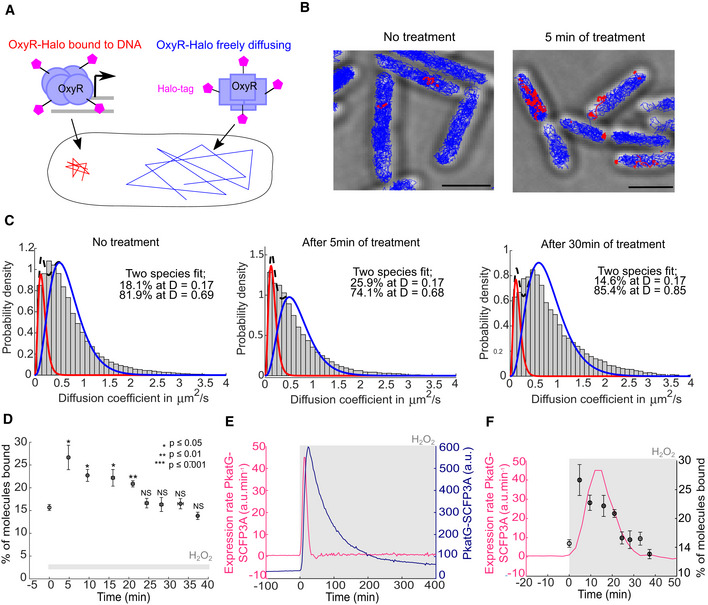
OxyR induces target genes within < 5 min after the start of H_2_O_2_ treatment Schematic of single‐molecule tracking to detect DNA‐binding of OxyR‐Halo based on the diffusion coefficient.Representative tracks of bound (red, diffusion coefficient (*D*) < 0.17 μm^2^/s) and diffusing OxyR‐Halo molecules (blue, *D* > 0.17 μm^2^/s) in untreated cells (left) and after 5 min of 100 μM H_2_O_2_ treatment (right). Scale bar 2 μm.Distribution of diffusion coefficients of OxyR‐Halo in untreated cells (Left, 83,271 tracks, three biological replicates), after 5 min of treatment (Middle, 27,213 tracks, three biological replicates) and after 30 min of treatment (Right, 16,186 tracks, three biological replicates) with 100 μM H_2_O_2_. Distributions were fitted to quantify the relative abundances of bound molecules (with average *D* = 0.17 μm^2^/s) and diffusing molecules (with average *D* ~ 0.68–0.85 μm^2^/s).Percentage of bound OxyR‐Halo molecules at different time points after 100 μM H_2_O_2_ treatment (mean ± SEM, three biological replicates). *P*‐values of the two‐sample *t*‐tests are indicated or not significant (NS). Stars are *P*‐values for test between OxyR‐Halo in untreated vs. treated cells at each time point.Expression rate of PkatG‐SCFP3A (pink) and PkatG‐SCFP3A intensity (dark blue) with 100 μM H_2_O_2_ treatment (988 cells, one biological replicate).Expression rate of PkatG‐SCFP3A (from panel E, pink, 988 cells, one biological replicate) and percentage of bound OxyR‐Halo molecules (from panel D, black, mean ± SEM, three biological replicates).

**Figure EV4:**
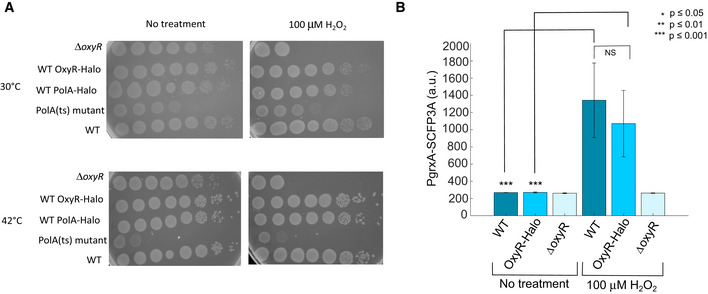
Survival assay with Pol1‐Halo and OxyR‐Halo fusions and induction of OxyR‐dependent gene expression confirms the functionality of the OxyR‐Halo fusion Survival assay without H_2_O_2_ treatment and with 100 μM H_2_O_2_ treatment performed at 30°C and at 42°C as PolA(ts) mutant is thermosensitive.Barplots of the average PgrxA‐SCFP3A intensity in WT, OxyR‐Halo and Δ*oxyR* mutant cells without treatment (mean ± SEM, four biological replicates, 3,748 cells for WT, 3,682 cells for WT OxyR‐Halo, 2,266 cells for Δ*oxyR*) and with 100 μM H_2_O_2_ treatment (mean ± SEM, four biological replicates, 3,106 cells for WT, 4,336 cells, for WT OxyR‐Halo, 2,816 cells for Δ*oxyR*). *P*‐values of the two‐sample *t*‐tests are indicated or NS (not significant).

Our observation that an adaptation delay causes a burst of mutations during H_2_O_2_ exposure led us to examine the timing of H_2_O_2_ sensing and signalling upstream of the gene expression response. For this, we developed a novel approach based on single‐molecule tracking of OxyR (Fig 3A and B). OxyR senses H_2_O_2_ via reversible oxidation that leads to the formation of a disulphide bond, which increases the binding affinity for target gene promoters to activate their transcription (Zheng *et al*, 1998; Lee *et al*, 2004). We reasoned that promoter binding should be detectable by measuring the mobility of OxyR in cells. To this end, we created a stable HaloTag fusion of OxyR for single‐molecule fluorescence imaging (Appendix Fig S8). The *E. coli* strain expressing OxyR‐Halo from the native endogenous locus showed the same tolerance to H_2_O_2_ as WT cells, in contrast to the hypersensitivity of a Δ*oxyR* mutant (Fig EV4A). Furthermore, the induction of the PgrxA‐SCFP3A reporter with 100 μM H_2_O_2_ was unaffected by the HaloTag fusion to OxyR (Fig EV4B). These tests demonstrate that the OxyR‐Halo fusion maintains full functionality. We performed single‐molecule tracking of OxyR‐Halo labelled with the cell‐permeable dye TMR (Banaz *et al*, 2019; Cassaro & Uphoff, 2022). In untreated cells, most OxyR molecules exhibited random diffusive motion, whereas H_2_O_2_ treatment led to the appearance of a distinct population of immobile molecules (Fig 3B and C). By measuring the diffusion coefficient (D), we quantified the fraction of immobile molecules relative to the diffusing pool of molecules as a readout for H_2_O_2_ sensing by OxyR. The immobile OxyR population peaked at 5 min after the start of H_2_O_2_ treatment, which was the earliest time point we were able to record with this method. Oxidised OxyR is deactivated via reduction by glutaredoxin 1 (Zheng *et al*, 1998), leading to a decay in the population of promoter‐bound OxyR when the scavenging system has lowered the intracellular H_2_O_2_ concentration (Fig 3C and D). The half‐life of the immobile OxyR population was ~15 min and OxyR binding returned to the basal level after ~30 min of constant H_2_O_2_ treatment, consistent with previous bulk measurements (Tao, 1999). To relate the dynamics of OxyR binding to the transcription of its target genes, we measured the PgrxA (Appendix Fig S9A) and PkatG (Fig 3D and E) promoter activities by computing the time‐derivatives of the SCFP3A reporter signals. The period of increased OxyR binding coincided with a spike in the promoter activities for both PgrxA (Appendix Fig S9B) and PkatG (Fig 3F). Together, these direct measurements show that maximal activation of OxyR occurs within a few minutes of constant H_2_O_2_ exposure and causes a rapid increase in the transcription rate of oxidative stress response genes.

### Constitutive OxyR response and priming adaptation prevent the mutagenesis burst

**Figure 4:**
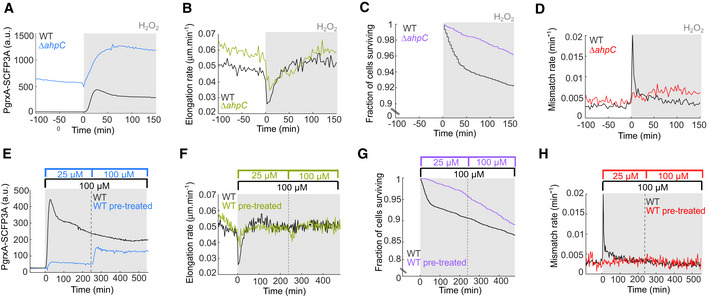
Constitutive OxyR response and priming adaptation prevent the mutagenic and toxic effects of sudden H_2_O_2_ exposure A–DOxidative stress response with 100 μM H_2_O_2_ treatment of Δ*ahpC* deletion mutant (coloured lines) compared with WT (black lines). (A) PgrxA‐SCFP3A intensity (1,433 cells, two biological replicates). (B) Cell elongation rate (3,015 cells, five biological replicates). (C) Fraction of cells surviving after start of treatment (2,795 cells, four biological replicates). (D) Rate of DNA mismatches per cell per minute (3,360 cells, five biological replicates).E–HOxidative stress response of WT cells pretreated with 25 μM H_2_O_2_ for 4 h followed by constant treatment with 100 μM H_2_O_2_. Response to 100 μM H_2_O_2_ without pretreatment is shown for comparison (black). (E) PgrxA‐SCFP3A intensity (605, one biological replicate). (F) Cell elongation rate (1,830 cells, three biological replicates). (G) Fraction of cells surviving after start of treatment (1,377 cells, two biological replicates). (H) Rate of DNA mismatches per cell per minute (2,020 cells, three biological replicates).

**Figure EV5:**
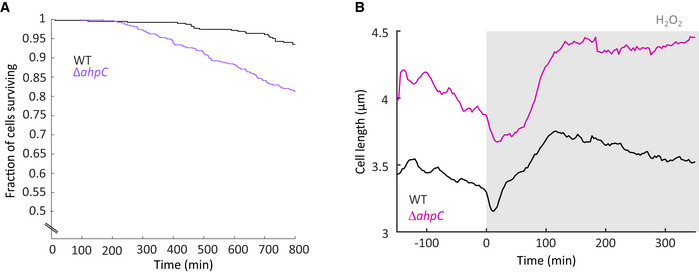
Growth characteristics of Δ*ahpC* mutant cells compared with WT cells Fraction of cells surviving without treatment for WT cells (black) and Δ*ahpC* mutant cells (purple, 528 cells, one biological replicate).The elongation rate of the Δ*ahpC* mutant is higher than for WT cells before and during 100 μM H_2_O_2_ treatment (Fig 4D). This can be explained by its higher cell length (B, pink, 3,360 cells, five biological replicates) compared with WT (black).

To test whether the transient delay of the OxyR response is indeed responsible for the sharp mutagenesis burst, we performed experiments under conditions in which the delay was abolished. We generated a strain with a constitutive OxyR response by elevating endogenous H_2_O_2_ levels with a deletion of the alkyl hydroperoxidase gene *ahpC* (Seaver & Imlay, 2001a). This strain showed increased basal expression of the PgrxA reporter before treatment (Fig 4A) and its elongation rate and survival were less affected after the addition of 100 μM H_2_O_2_ treatment than WT cells (Fig 4B and C). The mutagenesis burst was completely absent in Δ*ahpC* cells and the mismatch rate increased gradually during constant H_2_O_2_ treatment (Fig 4D). Hence, delayed OxyR response induction is responsible for the mutagenesis burst in WT cells. It is interesting to compare the behaviour of Δ*katG* and Δ*ahpC* strains in light of the distinct functions of the two H_2_O_2_ scavenging enzymes. Without treatment, Δ*ahpC* cells had a higher spontaneous death rate and increased cell length compared with wild‐type cells (Fig EV5A and B). Scavenging of endogenous H_2_O_2_ in untreated cells relies on the AhpCF, which has a lower enzymatic Km value than KatG (Mishra & Imlay, 2012). Indeed, whereas Δ*ahpC* deletion leads to the accumulation of endogenous H_2_O_2_ to an extent that the OxyR response becomes constitutively activated (Fig 4A), Δ*katG* deletion did not increase PgrxA reporter expression in untreated cells (Appendix Fig S10). Although KatG is dispensable in untreated cells, the higher Km value of the enzyme allows it to scavenge H_2_O_2_ at elevated concentrations when AhpCF becomes saturated (Mishra & Imlay, 2012). This important role of KatG is reflected in the rapid killing of Δ*katG* cells during 100 μM H_2_O_2_ treatment (Fig 2D), while the Δ*ahpC* mutant is largely tolerant to this dose (Fig 4C).

Exposing cells to sublethal concentrations of H_2_O_2_ is known to increase their survival of subsequent treatments (Imlay, 2008; Rodriguez‐Rojas *et al*, 2020). To test whether priming adaptation can also protect against the mutagenesis burst, we pretreated cells with a low dose of H_2_O_2_ (25 μM) for 4 h before exposure to the higher dose (100 μM). The pretreatment induced expression of the PgrxA reporter to an intermediate level (Fig 4E). Primed cells showed no reduction in elongation rate and no drop in survival when exposed to 100 μM H_2_O_2_ (Fig 4F and G). Moreover, the mutagenesis burst was abolished in primed cells (Fig 4H). Therefore, the lack of an adaptation delay due to a step‐wise increase in oxidative stress level is sufficient to avoid the mutagenesis burst. This is in agreement with a reduction in mutation frequency as seen by genome sequencing of primed cells treated with H_2_O_2_ (Rodriguez‐Rojas *et al*, 2020).

### DNA repair dynamics during oxidative stress adaptation

**Figure 5:**
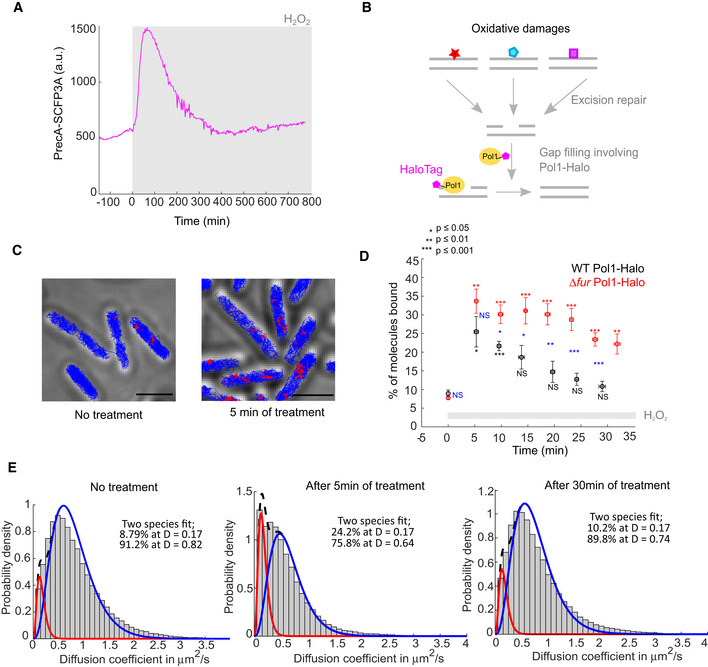
Dynamics of DNA damage response and DNA repair during oxidative stress adaptation SOS response reporter PrecA‐SCFP3A expression with 100 μM H_2_O_2_ treatment (861 cells, one biological replicate).Single‐molecule tracking of Pol1‐Halo as a reporter for the repair of oxidative DNA lesions. Immobile molecules reflect DNA‐bound Pol1.Representative tracks of bound (red, average *D* < 0.17 μm^2^/s) and diffusing Pol1‐Halo molecules (blue, *D* > 0.17 μm^2^/s) in untreated cells (left) and after 5 min of 100 μM H_2_O_2_ treatment (right). Scale bar 2 μm.Percentage of bound Pol1‐Halo molecules in non‐treated cells (time 0) and in response to 100 μM H_2_O_2_ treatment for WT cells (mean ± SEM, three biological replicates) and Δ*fur* mutant cells (mean ± SEM, four experiments biological replicates). *P*‐values of the two‐sample *t*‐tests are indicated or not significant (NS). Black stars are *P*‐values for test between Pol1‐Halo in untreated vs. treated cells at each time point. Red stars are *P*‐values for test between Pol1‐Halo Δ*fur* in untreated vs. treated cells at each time point. Blue stars are *P*‐values for test between Pol1‐Halo and Pol1‐Halo Δ*fur* at each time point.Distribution of diffusion coefficients of Pol1‐Halo without treatment (83,613 tracks, three biological replicates), and after 5 min (98,044 tracks, three biological replicates) and 30 min (112,780 tracks, three biological replicates) of 100 μM H_2_O_2_ treatment. Distributions were fitted to quantify the relative abundances of bound molecules (with average *D* = 0.17 μm^2^/s) and diffusing molecules (with average *D* ~ 0.64–0.82 μm^2^/s).

Oxidative DNA damage is not limited to mutagenic base lesions but also leads to single‐stranded and double‐stranded DNA breaks (Imlay, 2008). Our results indicate that the low rates of replication errors after an initial mutagenesis burst can be attributed to a reduction in intracellular H_2_O_2_ concentration following the upregulation of ROS‐scavenging enzymes. However, it is also plausible that DNA damage levels remain elevated even after adaptation but that DNA repair mechanisms efficiently remove mutagenic base lesions and strand breaks before they lead to replication errors. If so, DNA damage levels and DNA repair activities would be sustained after adaptation. To test this, we measured the expression dynamics of the SOS response, which is triggered by DNA breaks (Kreuzer, 2013). H_2_O_2_ treatment induced the expression of a PrecA‐SCFP3A reporter for the SOS response (Fig 5A). However, after an initial pulse of expression at 100 min, the reporter signal returned to the basal level after ~4–5 h of constant treatment. Therefore, DNA damage levels do not remain elevated after adaptation.

Different types of oxidative DNA lesions are targeted by a variety of DNA repair enzymes via the BER and Nucleotide Excision Repair (NER) pathways. To directly measure the timing of the overall repair of oxidative lesions during adaptation to H_2_O_2_, we chose DNA polymerase I (Pol1) as a universal reporter because it acts on DNA gaps, which are a common intermediate in both BER and NER (Friedberg, 2005) (Fig 5B). Single‐molecule tracking of a functional Pol1‐Halo fusion (Uphoff *et al*, 2013; Banaz *et al*, 2019) (Fig EV4A) showed that the population of immobile molecules increased after H_2_O_2_ treatment, with the highest binding activity seen at the earliest recorded time point (5 min; Fig 5C–E). Pol1 did not show sustained binding activity during constant H_2_O_2_ treatment (Fig 5C–E). Together, these results demonstrate that the induction of scavenging enzymes effectively protects cells against H_2_O_2_ present in their environment, reducing the formation of oxidative DNA damage down to the basal level within 30 min of continuous oxidative stress. This explains observations from genetic studies that the deletion of BER and NER enzymes does not sensitise cells to killing by H_2_O_2_ (Hoff *et al*, 2021).

### Perturbation of intracellular iron levels modulates basal mutagenesis and the mutagenesis burst induced by H_2_O_2_

**Figure 6:**
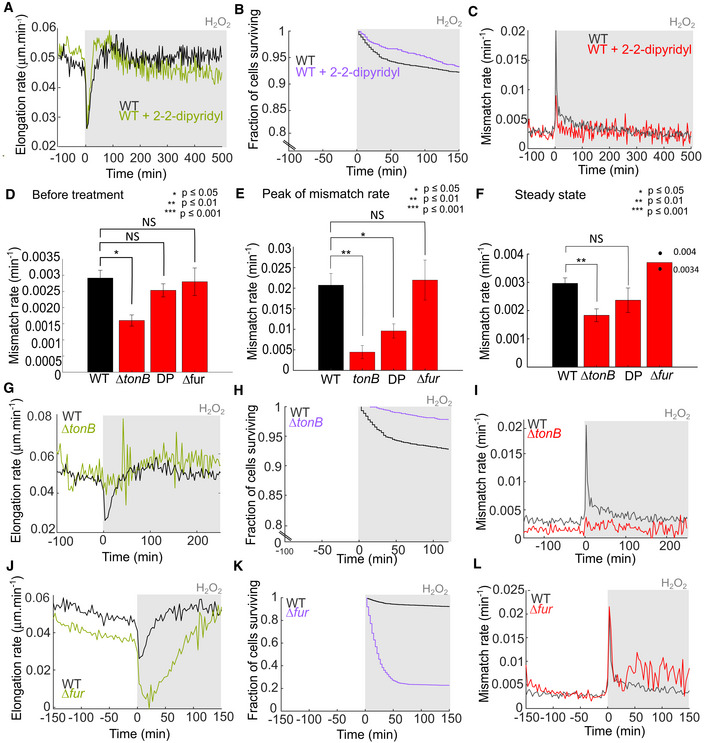
Perturbation of iron homeostasis impacts mutagenesis and adaptation to constant H_2_O_2_ treatment A–CWT cells with 100 μM H_2_O_2_ treatment in medium supplemented with 50 μM 2‐2‐dipyridyl (DP, coloured lines) or without supplement (black). (A) Cell elongation rate (2,081 cells, three biological replicates). (B) Fraction of cells surviving (1,104 cells, two biological replicates). (C) Rate of DNA mismatches per cell per minute (2,270 cells, three biological replicates).DBarplots showing the average mismatch rate per cell per minute for WT (black, seven biological replicates), DP (red, three biological replicates), Δ*tonB* (red, three biological replicates), Δ*fur* cells (red, four biological replicates) before treatment (mean ± SEM). Stars indicated the *P*‐value of the two‐sample *t*‐test.EBarplots showing the average mismatch rate per cell per minute for WT (black, eight biological replicates), DP (red, three biological replicates), Δ*tonB* (red, three biological replicates), Δ*fur* cells (red, four biological replicates), at the maximum of the mismatch rate peak with 100 μM H_2_O_2_ treatment (mean ± SEM). For Δ*tonB* the value was chosen as the average mismatch rate per cell per minute at the time corresponding to the average mismatch peak in WT cells. Stars indicated the *P*‐value of the two‐sample *t*‐test.FBarplots showing the average mismatch rate per cell per min for WT (black, eight biological replicates), DP (red, three biological replicates), Δ*tonB* (red, four biological replicates), Δ*fur* cells (red, two biological replicates) at the steady‐state after the initial mutation burst during constant treatment with 100 μM H_2_O_2_ (mean ± SEM). Stars indicated the *P*‐value of the two‐sample *t*‐test. For Δ*fur* cells, the two dots indicate the value obtained for each replicate.G–ISame as panels (A–C) for *tonB* deletion mutant (Δ*tonB*, coloured lines) and WT cells (black) with 100 μM H_2_O_2_ treatment. (G) Cell elongation rate (1,523 cells, three biological replicates). (H) Fraction of cells surviving (1,758 cells, three biological replicates). (I) Rate of DNA mismatches per cell per minute (1,733 cells, three biological replicates).J–LSame as panels (A–C) for *fur* deletion mutant (Δ*fur*, coloured lines) and WT (black) treated with 100 μM H_2_O_2_. (J) Cell elongation rate (2,593 cells, four biological replicates). (K) Fraction of cells surviving (3,067 cells, four biological replicates). (L) Rate of DNA mismatches per cell per minute (2,873 cells, four biological replicates).

The toxic and mutagenic effects of H_2_O_2_ are attributed to the Fenton reaction, which couples the oxidation of ferrous iron to the generation of highly reactive hydroxyl radicals (Imlay, 2008). Consequently, the regulation of iron homeostasis is a central feature of oxidative stress responses (Troxell & Hassan, 2013). To test the importance of iron for the observed mutagenesis burst, we added the iron chelator 2,2′‐dipyridyl (DP) to the growth media before and during H_2_O_2_ treatment (Fig 6A–C). We still observed a transient reduction in cell elongation after H_2_O_2_ addition, but DP accelerated the adaptation and return to normal growth (Fig 6A). However, the magnitude of the mutagenesis burst and the effect of H_2_O_2_ on cell mortality were both significantly reduced by DP (Fig 6B–F). Next, to decrease intracellular iron levels, we deleted the *tonB* gene, which is required for iron import (Fig 6G–I; Chakraborty *et al*, 2007). This mutant had the same generation time (Appendix Fig S11) and elongation rate (Fig 6G) as the WT but exhibited a significantly reduced rate of DNA mismatches even before treatment (Fig 6D and I), showing that intracellular iron contributes substantially to spontaneous mutation in optimal growth conditions. Furthermore, the mutagenesis burst was completely absent in Δ*tonB* cells treated with H_2_O_2_ (Fig 6E and I), and we saw no reduction in growth or survival after treatment (Fig 6G and H). However, the OxyR response was induced to the same level in WT, Δ*tonB* and DP‐treated cells, confirming that inhibition of the Fenton reaction does not affect H_2_O_2_ levels and oxidative stress response signalling (Appendix Fig S12A and B). TonB deletion had a stronger effect than DP treatment because we used a moderate concentration of DP that likely reduces but not completely depletes free iron in order to maintain normal cell growth rates (Appendix Fig S13; Rao & Kuzminov, 2022). To probe whether mutagenesis and toxic effects were limited by the amount of intracellular iron, we increased iron import by deleting the *fur* gene (Fig 6J–L; Touati *et al*, 1995). Cell elongation and survival were more strongly attenuated during the adaptation delay for Δ*fur* cells (Fig 6J and K). Although the height of the mutagenesis burst was unaffected by *fur* deletion, mismatch rates remained elevated postadaptation (Fig 6E, F and L) and the OxyR response was induced to a higher level than in WT cells (Appendix Fig S14). Furthermore, the DNA repair activity of Pol1‐Halo was increased and prolonged in the Δ*fur* mutant (Fig 5D).

## Discussion

ROS plays a key role in mediating antibacterial functions of the immune system (Fang *et al*, 2016) and during competition between bacterial species (Dong *et al*, 2015). It also has been suggested that ROS are produced when bacteria are treated with antibiotics (Kohanski *et al*, 2010b; Giroux *et al*, 2017; Hong *et al*, 2019). The ability of bacteria to survive elevated ROS levels relies on oxidative stress responses, which induce conserved scavenging enzymes that provide effective resistance to ROS such as H_2_O_2_. Nevertheless, the sublethal concentrations of H_2_O_2_ that occur frequently in the environment (Imlay, 2019) are still highly mutagenic even in wild‐type bacteria with functional oxidative stress responses (Bjelland & Seeberg, 2003; Rodriguez‐Rojas *et al*, 2020), indicating an inherent vulnerability in the bacterial ROS defence mechanisms. Here, we set out to identify the nature of this weakness. Specifically, we asked what the chronology of H_2_O_2_‐induced mutagenesis is in relation to the timing of cell killing and to the timing of the oxidative stress response in *E. coli*. We utilised microfluidic growth chambers to monitor intrinsic cellular dynamics while the environmental conditions and H_2_O_2_ concentrations were held constant.

We found that the OxyR response is very effective at preventing oxidative DNA damage and lethality, but the delay before the induction of H_2_O_2_ scavenging enzymes leaves cells vulnerable to the mutagenic effects of H_2_O_2_. Indeed, repeated H_2_O_2_ treatment leads to the selection of mutants with a constitutively active OxyR response (Anand *et al*, 2020), which avoids the toxicity and mutagenesis caused by the adaptation delay (Fig 4). In wild‐type cells during the adaptation delay, DNA repair pathways are actively removing oxidative DNA damage, but some of these lesions escape repair and become fixed as mutations. Our findings suggest that such mutation dynamics are not unique to H_2_O_2_ stress. We previously showed that continuous alkylation stress also triggers a mutagenesis burst that is terminated when the expression of DNA repair enzymes is upregulated in cells by the Ada protein (Uphoff, 2018). The mutagenesis bursts caused by oxidative stress and alkylation stress are fundamentally a consequence of adaptation delays, although the underlying molecular mechanisms are different. Whereas OxyR senses H_2_O_2_ within minutes and reliably activates stress response genes, the very low number of Ada proteins that is present in cells for sensing alkylation damage delays the induction of DNA repair genes over multiple cell generations (Uphoff *et al*, 2013; Uphoff, 2018). Different from oxidative stress, cells adapted to alkylation stress still experience high levels of DNA damage, as shown by continuous elevated expression of the SOS response and sustained BER activity (Uphoff *et al*, 2013; Uphoff, 2018). However, once activated, these repair mechanisms are effective at removing mutagenic lesions such that the mutation burst is restricted to the period of the adaptation delay when the DNA repair capacity is low.

Our findings underline the notion that the induction of reactive oxygen species as antibacterial agents is a double‐edged sword. A brief period of accelerated mutation supply could speed up the evolution of antibiotic resistance and host adaptation. We speculate that a burst of mutations increases the chance of evolutionary rescue when abrupt stress threatens a population to decline to extinction (Bell, 2017). However, it can take several generations before the expression of a new mutation is sufficient to confer stress resistance – a phenomenon known as phenotypic lag (Sun *et al*, 2018). In this case, stress responses can provide temporary tolerance for new mutants to survive the phenotypic lag. With live imaging, it is possible to administer defined stress treatments such as antibiotics via microfluidics and to monitor mutation supply, stress responses, cell fitness and population dynamics simultaneously. This approach complements genetic and population‐scale studies to provide direct insights into evolutionary mechanisms.

## Materials and Methods

### Reagents and Tools table

**Table:** 

Reagent/Resource	Reference or Source	Identifier or Catalog Number
**Experimental Models**		
SU000	AB1157	
SU471	AB1157, PolA‐11aa‐Halo::kan	This study/Banaz *et al* (2019)
SU620	AB1157, Δ*flhD*, Tn7::mkate2, mutL‐mYpet, carrying pUA066‐PkatG‐SCFP3A::kan (PSU033)	This study
SU621	AB1157, Δ*flhD*, Tn7::mKate2, mutL‐mYPet, carrying pUA139 PrecA‐SCFP3A::Kan (PSU032)	This study
SU699	AB1157, OxyR‐11aa‐Halo::kan	This study
SU718	AB1157, PolA‐11aa‐Halo, Δ*fur::kan*	This study
SU777	AB1157, Δ*flhD*, Tn7::mKate2, mutL‐mYPet, carrying pUA139 PgrxA‐SCFP3A::Kan (PSU044)	This study
SU778	AB1157, Δ*flhD*, Tn7::mkate2, mutL‐mYPet, Δ*katG* carrying pUA139‐PgrxA‐SCFP3A::kan (PSU044)	This study
SU779	AB1157, Δ*flhD*, Tn7::mKate2, mutL‐mYPet, Δ*ahpC* carrying pUA139 PgrxA‐SCFP3A::kan (PSU044)	This study
SU780	AB1157, Δ*flhD*, Tn7::mKate2, mutL‐mYPet, Δ*fur* carrying pUA139 PgrxA‐SCFP3A::kan (PSU044)	This study
SU802	AB1157, Δ*flhD*, Tn7::mkate2, mutL‐mYpet, Δ*oxyR*, carrying pUA139 PgrxA‐SCFP3A::kan (PSU044)	This study
SU803	AB1157, Δ*flhD*, Tn7::mKate2, mutL‐mYPet, Δ*tonB* carrying pUA139 PgrxA‐SCFP3A::kan (PSU044)	This study
SU786	AB1157, OxyR‐Halo (SU701) + pUA139 Pgrxa‐SCFP3A kan (PSU044)	This study
SU887	AB1157 pUA139‐Pgrxa‐SCFP3A::kan (pSU044)	This study
SU888	AB1157 Δ*oxyR*‐ pUA139 Pgrxa‐SCFP3A::kan (pSU044)	This study
SU1072	MG1655 Δ*flhD‐*Tn7::mkate2, mutL‐mYPet + pUA139‐PgrxA‐SCFP3A Kan (PSU044) kan	This study
CM5255	polA4113(ts) mutation	CGSC
**Recombinant DNA**		
pSU044	pUA139 PgrxA‐SCFP3A kan	This study
pSU033	pUA066‐PkatG‐SCFP3A kan	This study
pSU032	pUA139‐PrecA‐SCFP3A kan	This study
pSU005	R6kgamma ori 11aa Halo frt kan frt	Banaz *et al* (2019)
**Oligonucleotides and sequence‐based reagents**		
SU207_KatG‐F ‐ for checking ΔkatG deletion	CGC ATC CGT GGA TTA ATT	This study
SU252_katG‐R ‐ for checking ΔkatG deletion	ACTTCGTGTTGACCTGTT	This study
SU237_AhpCdel_fwd ‐ for checking ΔahpC deletion	TCGCCGCTGGCGGTGCAA	This study
SU238_AhpCdel_rev ‐ for checking ΔahpC deletion	GCG TCA GAG CAA GGC GGC	This study
SU239_TonBdel_fwd ‐ for checking ΔtonB deletion	TCA CTG ATC CTG ATC GTC	This study
SU240_TonBdel_rev ‐ for checking ΔtonB deletion	GCA ACG CTA TAA AGC GAC	This study
SU241_Furdel_fwd ‐ for checking Δfur deletion	TGC CAG GGA CTT GTG GTT	This study
SU242_Furdel_rev ‐ for checking Δfur deletion	ATC AGG CGG TGA AAG CCG	This study
SU328_OxyR‐Lred ‐ for deletion of OxyR gene by lambda red	ctattctacctatcgccatgaactatcgtggcgatggaggatggataATGGGAATTAGCCATGGTCC	This study
SU329_OxyR‐Lred ‐ for deletion of OxyR gene by lambda red	gacgatggcggaagcctatcgggtagctgcgttaaacggtGTGTAGGCTGGAGCTGCTTCG	This study
SU231_OxyRdel_fwd ‐ for checking ΔoxyR deletion	TCT CGA AAC GGG CAG TGA	This study
SU232_OxyRdel_rev ‐ for checking ΔoxyR deletion	TCG GTC AGG CGA TTA TGG	This study
SU306_OxyR_f ‐ for fusion of Halo tag to OxyR	TCCGCGCAAGAATGGATGGCCATTTCGATAAAGTTTTAAAACAGGCGGTTTCGGCTGGCTCCGCTGC	This study
SU307_OxyR_r ‐ for fusion of Halo tag to OxyR	CGGAAGCCTATCGGGTAGCTGCGTTAAACGGTTTAAACCGCCTGTTTTAATATGAATATCCTCCTTAG	This study
SU308_OxyR (pcr test)_f ‐ for checking OxyR‐Halo fusion	GTTTATCTGCCGTGCATTAAGCC	This study
SU309_OxyR_(pcr test)_r ‐ for checking OxyR‐Halo fusion	AAGAGATTCTGGGTATTCACTGC	This study
SU188_Pol1Halo_LRed ‐ for fusion of Halo tag to Pol1‐Halo	TGCCGTTGCTGGTGGAAGTGGGGAGTGGCGAAAACTGGGATCAGGCGCACTCGGCTGGCTCCGCTGC	This study
SU189_Pol1Halo_Lred ‐ for fusion of Halo tag to Pol1‐Halo	ACGTGACAGCTTATGTTGCTTACTTACGAAAAAAGGCATGTTCAGGCGAATCTATGAATATCCTCCTTAG	This study
**Chemicals, enzymes and other reagents**		
SOC medium	NEB B9020S	New England Biolabs
M9 minimal salts 5×	M9956	Sigma
Solution 10× amino acids	11130‐036	Gibco
L‐Proline	A3453,0100	Biochemica
Thiamine	A0955,0050	Biochemica
Pluronic F‐127	P2443‐250G	Sigma
Dow Corning Sylgard 184 kit	1667370	Farnell electronics
1H,1H,2H,2H‐perfluorooctyl silane	448931	Sigma
Coverslips No 1.5 24 × 50 mm	631‐0147	VWR
Tygon Microbore silicon tubing	ND 100‐80	VWR
30% W/W solution of H_2_O_2_	H1009‐100mL	Sigma
2,2‐Dipyridyl	D216305	Sigma
Catalase	C9322	Sigma
TMR dye	G8251	Promega
SDS–PAGE gels	XP00102	Invitrogen
PageRuler Plus Prestained Protein Ladder 10 to 25	26619	Thermo scientific
**Software**		
Matlab R2020b	https://uk.mathworks.com/products/matlab.html	For analysis of microfluidic and single molecule tracking data
NIS element imaging software ‐ V4.30.02 (64 bit)	Nikon	For microfluidic microscopy
Andor SOLIS X‐1000 (64bit) – Solis version 4.31.30023.0	Andor	For single molecule tracking

### Methods and Protocols

#### Strains and plasmids construction

Unless otherwise indicated, strains were all derived from *E. coli* AB1157 and constructed using classic molecular biology and genetics techniques. For microfluidic microscopy experiments, we used a previously constructed strain, carrying a Δ*flhD* gene deletion to inhibit cell motility, a constitutively‐expressed fluorescent cell marker P_RNAI_‐mKate2 and an endogenous MutL‐mYPet fusion (Uphoff, 2018). It is known that *E. coli* AB1157 carries an amber mutation in *rpoS* (Visick & Clarke, 1997), which may affect oxidative stress adaptation. We thus confirmed our central observations in separate experiments using *E. coli* MG1655 as strain background (Appendix Fig S15A–C).

For single‐molecule tracking experiments, OxyR‐Halo and Pol1‐Halo fusions were constructed by lambda Red recombination (Datsenko & Wanner, 2000). We used a plasmid (pSU005, see plasmid list, Banaz *et al*, 2019) designed previously carrying an 11 amino acid linker (11 aa linker, SAGSAAGSGEF) followed by a HaloTag and kanamycin resistance gene encompassed by two Flp recombinase recognition target (Frt) sites. To amplify these elements, we used forward primers containing 50 nt overhangs homologous to the C‐terminal extremity of *oxyR*/*polA* gene and having the complementarity sequence to the 11 aa linker. The reverse primers had 50 nt homology to the sequence immediately downstream of *oxyR*/*polA* gene (Reagents and Tools table). We then inserted the PCR product on the chromosome using AB1157 cells containing the temperature‐sensitive pkD46 plasmid, which carries the lambda red recombination proteins controlled by an araBAD promoter. Cells grew in LB at 30°C to allow plasmid replication and 0.2% arabinose was added to the medium to induce the expression of the recombination proteins. When cells reached 0.6 OD600, several cycles of centrifugation and resuspension of the pellet were performed using dH_2_O and 10% glycerol. Fourteen microliter of PCR product were then transformed by electroporation in 90 μl cells (2.5 kV electric pulse for 5 ms, Bio‐Rad micropulser). Cells were recovered in SOC outgrowth medium (NEB B9020S) for 1 h at 37°C before selection on kanamycin plates. pKD46 plasmid was cured by streaking single colonies twice at 37°C. Insertion was confirmed by colony PCR and the allele was moved to WT AB1157 by P1 phage transduction.

All deletion mutants were moved to AB1157 by P1 transduction and antibiotic selection, using the corresponding Keio collection strains from the Coli Genetics Stock Center (CGSC; Baba *et al*, 2006) Δ*katG*::kan (CGSC 10827), Δ*ahpC*::kan (CGSC 8713), Δ*tonB*::kan (CGSC 11229), Δ*fur::kan* (CGSC 8758), Δ*mutS*::kan (CGSC 10126). All gene deletions were checked by PCR using primers flanking the gene (Reagents and Tools table). The antibiotic resistance flanked by frt sites was then removed using Flp recombinase expressed from pCP20 plasmid. We constructed the Δ*oxyR*∷kan mutant using lambda Red recombination and the plasmid pKD4 following the protocol from (Datsenko & Wanner, 2000) and as described above for the HaloTag fusions (Reagents and Tools table).

Reporter plasmids for the oxidative stress response were constructed based on pSC101 plasmids that expressed GFPmut2 under the control of PgrxA or PkatG (Zaslaver *et al*, 2006), The GFPmut2 was then replaced by the fast‐maturing CFP variant SCFP3A (Balleza *et al*, 2018) using Gibson assembly. Similarly, we constructed a PrecA‐SCFP3A reporter plasmid for the SOS response. All reporter plasmids were checked by sequencing and then inserted in different strains by heat‐shock transformation (Reagents and Tools table).

Before any microscopy experiment, strains were streaked from glycerol stocks stored at −80°C on LB plates with appropriate antibiotics and incubated overnight at 37°C.

#### Microfluidics experiments

##### Cell culture before microscopy

For microfluidic microscopy, a single colony was grown in LB at 37°C with shaking for 6–8 h, and the culture was diluted 1/50 overnight in 4 ml of M9 medium (M9 minimal salts 5× Sigma M9956) supplemented with 0.2% glucose, MEM amino acids (solution 10× Gibco 11130‐036), 0.1 mg/ml L‐proline (Biochemica A3453,0100), 0.5 μg/ml thiamine (Biochemica A0955,0050), 2 mM MgSO_4_, 0.1 mM CaCl_2_ and the appropriate antibiotic. The next morning, overnight cultures were diluted 1/50 in 4 ml of the same M9 supplemented medium with antibiotic and incubated with shaking for 3 h at 37°C before loading in microfluidics devices. 0.85 mg/ml of surfactant pluronic F127 (Sigma, P2443‐250G) was added to the morning culture to avoid cell aggregation in the device.

##### Preparation of the devices

Microfluidic experiments were performed with the “mother machine” type of devices (Wang *et al*, 2010). Media is flowed in channels of sizes 100 × 25 μm (width × depth) and bacteria are in growth trenches of dimensions 1.2 × 1.2 × 20 μm (width × depth × length). These devices were prepared as described by Moolman *et al* (2013) and Uphoff (2018). Devices were made in Polydimethylsiloxane (PDMS) from a silicon wafer (produced by Kavli Nanolabs, Delft University) in two steps. An intermediate negative PDMS mould was made from the silicon wafer, by mixing monomer and curing agent 1:5 (Dow Corning Sylgard 184 kit (Farnell electronics, 1667370)). After removing bubbles in vacuum, the intermediate mould was cured at 65°C for 2 h and subsequently treated overnight with Trichloro (1H,1H,2H,2H‐perfluorooctyl)silane (Sigma 448931). The next day, after washing the intermediate mould with 100% ethanol, PDMS devices were made with a 1:10 mix of monomer and curing agent. Bubbles were removed in vacuum and curing was done at 65°C for 2 h. To make the preparation of the devices easier, we subsequently obtained silicon wafer from a different supplier (Conscience). In this case, the wafer had a negative relief, which means that PDMS devices could be made in one step (without an intermediate PDMS mould).

Before each experiment, one device was cut with a scalpel and holes for media inlet and outlet were inserted using a 0.75 mm biopsy punch. The device was then washed with 100% ethanol and dried with nitrogen gas three times. Microscope coverslips (No 1.5 24 × 50 mm, VWR 631‐0147) were cleaned before bonding to the PDMS device. First coverslips were sonicated in acetone for 20 min, washed twice with dH_2_0, followed by 20 min of sonication in 100% isopropanol and drying with nitrogen gas. Coverslips and devices were then bonded by treatment in air plasma (Plasma Etch PE‐50) followed by 30 min in an oven at 95°C.

One milliliter of the morning culture was concentrated by centrifugation and resuspended in 100 μl supernatant. Cells were then loaded in the PDMS device, by pipetting in the inlet/outlet holes. The whole device was then centrifuged for 10 min at 2,348 *g* in a benchtop centrifuge with a custom holder to push the cells into the growth trenches. Supplemented M9 containing 0.85 mg/ml pluronic F127 without antibiotics was flowed via Tygon Microbore silicon tubing (VWR ND 100‐80/0.508*1.524) that linked the device to a syringe pump (ALADDIN‐220, World Precision Instruments) used with 30 ml syringes. Two syringes on two identical pumps were used for the experiment, both containing supplemented M9 and pluronic, and one of the two containing H_2_O_2_ (Sigma, 30% W/W solution H1009‐100 ml) at the concentrations stated in the text and figures. The tubing from the two syringes was linked to the same device via a Y‐junction with clamps to avoid backflow. During the first 10 min after loading, media was pumped at a rate of 2.5 ml/h to flush out cells that were outside growth trenches and the flow rate was subsequently lowered to 0.5 ml/h for data acquisition. Cells grew for 1 h in trenches before starting data acquisition to equilibrate to the environment. Data were acquired on average for 3 h before switching to a medium containing H_2_O_2_. Just after the start of the treatment the flow rate was increased for 10 min to 2.5 ml/h to ensure rapid exchange of growth media and then lowered to 0.5 ml/h for the remainder of the experiment. When indicated, 0.005 mg/ml 2,2'dipyridyl (DP, Sigma D216305, dissolved in DMSO) was added to supplemented M9 in both syringes (without and with H_2_O_2_). Catalase (Sigma C9322, 1 μg/ml) was added to the morning culture of the Δ*oxyR* mutant strain to help with the growth and loading of the cells in the microfluidic chip. Catalase was only added in the culture until the loading of the chip and not in the media used during data collection.

##### Imaging

Microfluidics experiments were performed on a Nikon Ti Eclipse inverted fluorescence microscope with a perfect focus system, oil immersion objective 100× NA1.4, motorised stage, sCMOS camera (Hamamatsu Flash 4) and LED excitation source (Lumencore Spectra X). The temperature chamber (Okolabs) allowed performing the experiments at 37°C. We recorded time‐lapse movies with a frame rate of 1/3 min using NIS‐Element software (Nikon) across three spectral channels (LED excitation wavelengths λ: 555, 508, 440 nm) to image mKate2, MutL‐mYpet and CFP reporters, respectively. One image is acquired from each channel consecutively using exposure times of 100 ms (λ = 555 nm), 300 ms (λ = 508 nm) and 75 ms (λ = 440 nm); LED intensity for all channels was 50% maximal output. One single triband dichroic and three separate emission filters were used to separate excitation and emission light. Up to 48 fields of view can be imaged across the microfluidic device within the 3‐min time‐lapse window. On average, 20 channels with bacteria were present per field of view such that about ~1,000 mother cell traces were recorded per experiment.

##### Image analysis

Time‐lapse movies were analysed using custom scripts written in MATLAB (Mathworks). The shape of the mother cell at the closed end of each trench was automatically segmented based on the constitutive cytoplasmic mKate2 signal as described in Norman *et al* (2013). Cell length was calculated based on the segmentation mask and cell division events were identified as sharp drops in cell length. The generation time corresponds to the time interval between consecutive division events. The cell elongation rate was calculated from the difference in cell length per time interval. During H_2_O_2_ treatment, cell traces were truncated manually when cells ceased elongation and did not divide again until the end of the experiment. Filamentous cells escape from the growth trenches when their length reaches the open end. Cell traces of filamentous cells were manually truncated after their last division. For cells that lysed, traces were truncated automatically by the segmentation and lineage tracing algorithm. We quantified cell survival during H_2_O_2_ treatment as the fraction of cell traces before truncation relative to the number of cell traces at the start of the treatment. CFP reporter intensities were computed from the average CFP pixel intensities inside the segmentation mask area per cell, after subtracting the camera background signal outside of cells. To account for the fluorescence maturation time of SCFP3A at 37°C, we deconvoluted the observed intensity signal using an exponential function kernel with a decay constant of 6.4 min (Balleza *et al*, 2018). All intensity traces in the figures show the deconvoluted data. A spot‐finding algorithm was used to detect MutL‐mYPet foci, using a 3‐pixel Gaussian band‐pass filter and intensity thresholding. The cell‐average mismatch rate per cell per minute corresponds to the total number of MutL‐mYPet foci divided by the number of observed cells in each frame and expressed in units of events/min based on the time interval of 3 min between frames. If the same mismatch event was detected in several consecutive frames, the event was counted only for the first frame. Plots of mismatch rate per cell per minute, CFP intensity, cell length, elongation rate, etc versus time show the average value at each time point across all cells, typically from three or more independent experiments performed on different days. Data from independent experiments were aligned temporally postacquisition based on the time of H_2_O_2_ addition. Promoter activities of the gene expression reporters were computed from the difference in total CFP intensity per time interval, divided by the segmented cell area.

#### Single‐molecule tracking

##### Functionality of HaloTag fusion proteins

The functionality of OxyR and Pol1 fusions to the HaloTag was tested by different assays detailed below.

##### H_2_O_2_ sensitivity assay

Ten‐fold serial dilutions of the analysed strains were spotted on LB‐agar plates containing no H_2_O_2_ or 100 μM H_2_O_2_. Two sets of plates were made and incubated, respectively, at 30 and 42°C as a control for the temperature‐sensitive *polA*(ts) mutant strain (Fig EV4A).

##### Expression of OxyR‐regulated fluorescent reporter

To assess the functionality of the OxyR‐Halo fusion, we analysed the induction of the OxyR response using a plasmid carrying PgrxA‐SCFP3A as a fluorescent transcriptional reporter. For consistency, we prepared cells from the WT AB1157 and OxyR‐Halo fusion strains following the same procedure as for single‐molecule tracking experiments (detailed below) but omitted the TMR dye to avoid cross‐talk with the CFP reporter. Cells were treated by adding 100 μM H_2_O_2_ 40 min prior to data acquisition. Cells were immobilised on agarose gel pads (described below) and CFP intensities were measured on the Nikon TiE system (described above) using 50% LED intensity at 100 ms exposure time. The CFP intensity per segmented cell area was obtained by segmentation of cell shapes from a phase contrast snapshot (described below) using MATLAB (Fig EV4B).

##### Protein gel

To confirm that HaloTag fusions are of the expected molecular weight and not degraded, we performed SDS–PAGE in‐gel fluorescence measurements. Strains were grown and labelled with TMR dye as for single‐molecule tracking experiments but with only one wash of medium to remove free dye (as opposed to four washes for single‐molecule imaging). After labelling, the OD600 of the culture was adjusted to 0.05. One milliliter of culture was centrifuged, resuspended in 15 μl of 2× SDS loading buffer +15 μl of dH_2_O and incubated for 10 min at 95°C. Fifteen microliter of sample was loaded on precasted SDS–PAGE gel (Invitrogen XP00102) with protein ladder (PageRuler Plus Prestained Protein Ladder 10 to 25, Life Technologies 26619) and run for 1.5 h. The gel was imaged using 532 nm laser illumination (Fujifilm Typhoon FLA 7000; Appendix Fig S8).

##### Cell culture and labelling

For single‐molecule imaging, a single colony was grown in LB with appropriate antibiotic for 6–8 h the day before the experiment. Eight microliter of culture were diluted in 4 ml of supplemented M9 medium and incubated overnight with the appropriate antibiotic. The next morning the culture was diluted 1/50 and incubated for 2 h with shaking at 37°C. One milliliter of culture was concentrated to 100 μl before labelling the Halotag as described in Banaz *et al* (2019) and Cassaro & Uphoff (2022). Briefly, 5 μl of 50 μM stock solution of TMR dye (Promega G8251) was added to the concentrated culture, mixed vigorously and incubated for 30 min at 25°C. The labelled culture was centrifuged and washed four times with 1 ml supplemented M9 medium to remove the free dye. Cells were then recovered at 37°C with shaking for 30 min and washed again, and 1 μl of concentrated cell suspension was pipetted on an agarose pad (1% agarose in supplemented M9 medium, with or without 100 μM H_2_O_2_) and sandwiched between two coverslips. Imaging was started as soon as possible after cells were placed on the pads (within ~5 min).

##### Data acquisition

Single‐molecule tracking was performed using a custom‐build Total Internal Reflection Fluorescence Microscope (TIRF) under oblique illumination (Wegel *et al*, 2016; Banaz *et al*, 2019) and controlled via Andor Solis and Toptica iChrome software. The microscope was constructed using ASI Modular Infinity Microscope components (MIM and RAMM, ASI). A motorised XY stage (MS2000 closed‐loop with linear encoders, ASI) with a Z‐piezo insert (PZ‐2150, ASI) was operated by a stage and focus controller (ASI). The 100× NA 1.40 oil immersion objective (UPLSAPO, Olympus) was attached to a linear translation stage (LS50, ASI) for coarse focusing. Excitation light was provided by a multilaser engine (iChrome MLE, Toptica) with 405, 488, 561 and 640 nm lasers. Laser light exiting the optical fibre was collimated with a 150 mm visible achromatic lens (Thorlabs) and focused into the objective back‐focal plane with a 200 mm visible achromatic lens (Thorlabs). Laser excitation angle could be continuously adjusted between brightfield and TIRF by moving the fibre mount and lenses perpendicular to the beam direction on a linear translation mount (Thorlabs). Excitation and emission light were separated using a multi‐band dichroic mirror (zt405/488/561/640rpc, Chroma) and fluorescence emission filter (zet405/488/561/640 m, Chroma) that were mounted in a filtercube (91041, Olympus). Fluorescence light was focused onto an EMCCD camera (iXon 897 Ultra, Andor) using a 300 mm achromatic tube lens module (ASI) giving a total magnification of 167×, equivalent to an image pixel size of 96 nm. For transmitted light illumination was provided by an LED light source (ASI) mounted on an Olympus IX2 condenser.

Movies of 10,000 frames with 15.48 ms time interval between frames were acquired under constant 561 nm excitation laser illumination at 0.5 kW/cm^2^. This induces stochastic blinking of TMR fluorophores such that only a small subset of labelled molecules is fluorescent at any time. Each field of view was exposed to 561 nm constant laser illumination at 0.5 kW/cm^2^ for about 30 s before acquisition to deactivate the majority of TMR fluorophores, providing a suitable density of fluorescent spots for single‐molecule detection and tracking. The acquisition time of each movie was noted relative to the time since the cells had been placed on the agarose pad with H_2_O_2_ treatment and time points were taken approximately every 5 min. An image of each field of view was also acquired with transmitted light for cell segmentation. All single‐molecule tracking experiments were performed at room temperature and were repeated at least three times on different days for each condition.

##### Data analysis

Single‐molecule tracking data were analysed in MATLAB as previously described (Stracy *et al*, 2016).

Candidate positions of fluorescent molecules were identified using threshold‐based spot detection in the band pass‐filtered images, followed by subpixel localization using a phasor algorithm (Martens *et al*, 2018). Localizations that appear within a spatial window of 8 pixels in consecutive frames were linked to tracks (Crocker & Grier, 1996), with a memory parameter of 1 frame (allowing for blinking or missed detection of a molecule). Cell outlines were automatically segmented from transmitted light images using a modified version of MicrobeTracker (Sliusarenko *et al*, 2011) combined with SuperSegger (Stylianidou *et al*, 2016). Any tracks with a mean position outside a segmented cell area were excluded from the analysis. The mean diffusion coefficient (D) for each track inside cells was calculated from the mean squared displacement (MSD) averaged over four steps (5 frames): D = MSD/(4∙Δ*t*), with Δ*t* = 15.48 ms. Shorter tracks were discarded from the analysis and longer tracks were truncated to four steps. The distribution of diffusion coefficients was fitted with an analytical model of two species of molecules with diffusion coefficients *D*_1_ and *D*_2_ (Saxton, 1997):

$$f\left(x,D_{1},D_{2},A_{1},A_{2}\right)=\frac{A_{1}{\left(\frac{4}{D_{1}}\right)}^{4}x^{3}e^{-4x/D_{1}}}{6}+\frac{A_{2}{\left(\frac{4}{D_{2}}\right)}^{4}x^{3}e^{-4x/D_{2}}}{6}$$

The relative abundances of the two populations *A*_1_ and *A*_2_ were constrained with *A*_1_ + *A*_2_ = 1. Fitting the combined data from all measurements and time points yielded a mean diffusion coefficient of the immobile population of molecules of *D*_1_ = 0.17 μm^2^/s. This value was subsequently constrained for fitting the distributions of each time point post‐treatment separately. The value of *D*_2_ for the mobile molecules was left unconstrained. The percentage of the immobile population *A*_1_ at each time point was used for Figs 3D and 5D. Error bars represent the standard error of the mean (SEM) for the population percentage (on the *y* axis) and the time post‐treatment (on the *x* axis).

#### Rifampicin assay

Mutation frequency was measured using rifampicin resistance assays following the protocol from Rodriguez‐Rojas *et al* (2020) and adapting it to the conditions of this study (Fig EV3). Briefly, three cultures of WT cells were grown until OD ~0.2 in LB medium. At this time, the volume of each culture was increased to 50 ml and then divided into two tubes of 25 ml. The former was kept untreated, the latter was treated with 1 mM H_2_O_2_, and 5 ml of culture was collected at different treatment times. Treatment was stopped at 5, 12, 20, 27 and 35 min by adding 10 μg/ml of catalase (Sigma C9322) to the 5 ml of culture and by then centrifuging for 6 min at 2,588 *g*. Treated and untreated cultures were then concentrated in 1 ml LB and grown at 37°C overnight. The next morning, 100 μl of culture was plated on LB plates containing 100 μg/ml of rifampicin. Serial dilutions were performed, and 100 μl of a 10^−7^ dilution of each culture was plated on LB plates without rifampicin. We calculated mutation frequencies by dividing the number of colonies counted on LB plates containing rifampicin by the number of colonies on LB plates without rifampicin for each culture. For each condition, the experiment was performed on three separate days with three independent cultures per day. Mean mutant frequency and standard error of the mean for the nine total cultures were calculated and a two‐sample *t*‐test performed.

## Author contributions

**Valentine Lagage:** Conceptualization; resources; data curation; formal analysis; validation; investigation; visualization; methodology; writing – original draft; project administration; writing – review and editing. **Victor Chen:** Resources; data curation; investigation; methodology. **Stephan Uphoff:** Conceptualization; software; supervision; funding acquisition; methodology; writing – original draft; project administration; writing – review and editing.

## Disclosure and competing interests statement

The authors declare that they have no conflict of interest.

## Supporting information



Appendix

Expanded View Figures PDF

PDF+

## Data Availability

Data and analysis code associated with the study have been deposited in Oxford University Research Archive. Dataset: Lagage *et al*, 2022: Adaptation delay causes a burst of mutations in bacteria responding to oxidative stress: https://ora.ox.ac.uk/objects/uuid:16808a32‐c198‐4d70‐988c‐3b214e287ad8.
