# Supplementary material for: Adaptation delay causes a burst of mutations in bacteria responding to oxidative stress
Source: EMBO Rep. 2022 Nov 17;24(1):e55640. doi: 10.15252/embr.202255640 (PMC9827559; doi:10.15252/embr.202255640)
Supplement: Supplementary file 1 — Appendix [file EMBR-24-e55640-s004.pdf]

APPENDIX

APPENDIX SUPPLEMENTARY FIGURES……………………………………………………………………………… 2-16

Appendix Figure S1……………………………………………………………………………………………… 2

Appendix Figure S2……………………………………………………………………………………………… 3

Appendix Figure S3……………………………………………………………………………………………… 4

Appendix Figure S4……………………………………………………………………………………………… 5

Appendix Figure S5……………………………………………………………………………………………… 6

Appendix Figure S6……………………………………………………………………………………………… 7

Appendix Figure S7……………………………………………………………………………………………… 8

Appendix Figure S8……………………………………………………………………………………………… 9

Appendix Figure S9……………………………………………………………………………………………… 10

Appendix Figure S10…………………………………………………………………………………………… 11

Appendix Figure S11…………………………………………………………………………………………… 12

Appendix Figure S12…………………………………………………………………………………………… 13

Appendix Figure S13…………………………………………………………………………………………… 14

Appendix Figure S14…………………………………………………………………………………………… 15

Appendix Figure S15…………………………………………………………………………………………… 16

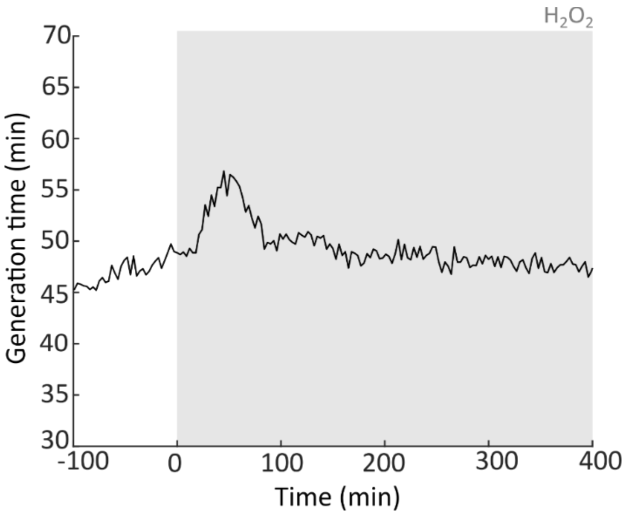

Appendix Figure S1 - Transient increase of the generation time in WT cells treated with H_2O_2.

Generation time of WT cells treated with 100 µM of H_2O_2 (6562 cells, 8 biological replicates).

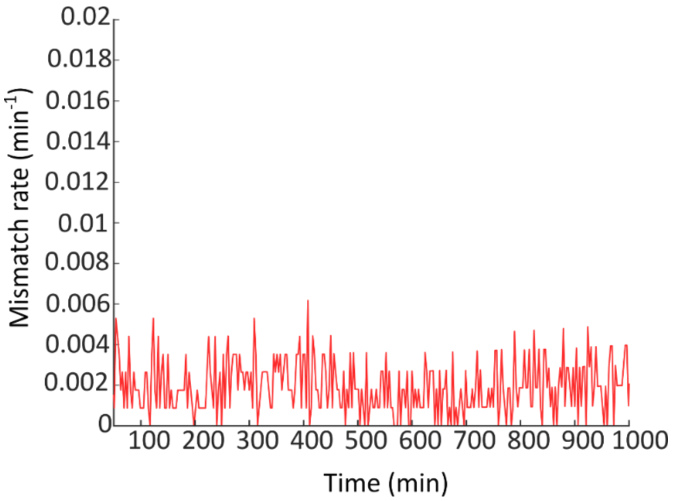

Appendix Figure S2 - Mismatch rate is stable over time in untreated cells.

Rate of DNA mismatches per cell per minute in untreated cells (651 cells, 1 biological replicate).

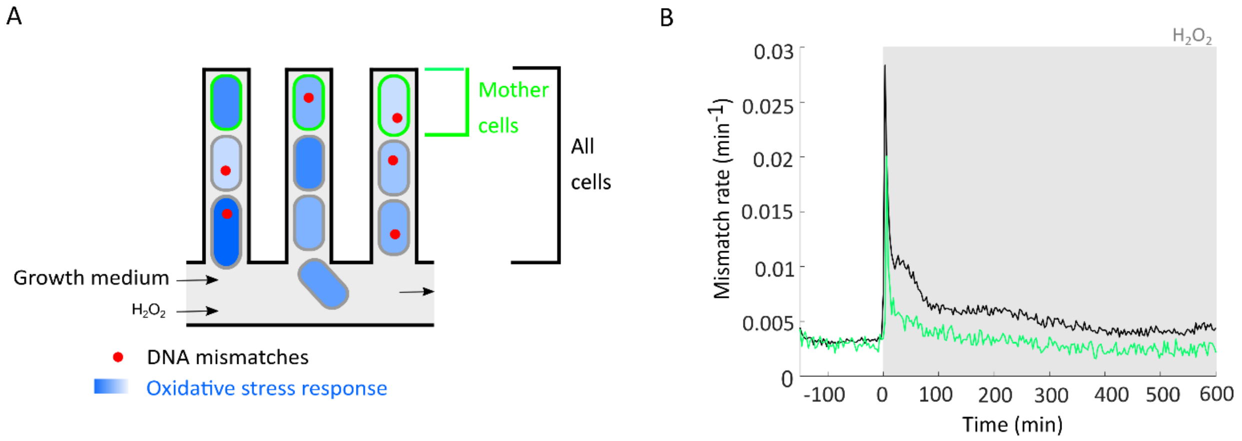

Appendix Figure S3 - All cells in the microfluidic growth trenches experience a burst of mutagenesis.

A Throughout the study, we focused our analysis on the mother cells that can be continuously observed at the closed end of each growth trench over multiple generations.

B Rate of DNA mismatches per cell per minute for all cells in the trenches with 100 µM H_2O_2 treatment (35082 cells, 8 biological replicates).

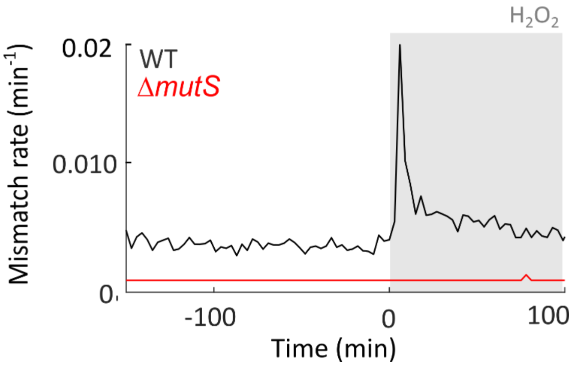

Appendix Figure S4 - No MutL-mYPet foci are detected in a Δ*mutS* deletion strain.

Rate of DNA mismatches per cell per minute of a Δ*mutS* mutant strain with 100 µM H_2O_2 treatment (551 cells, 1 biological replicate).

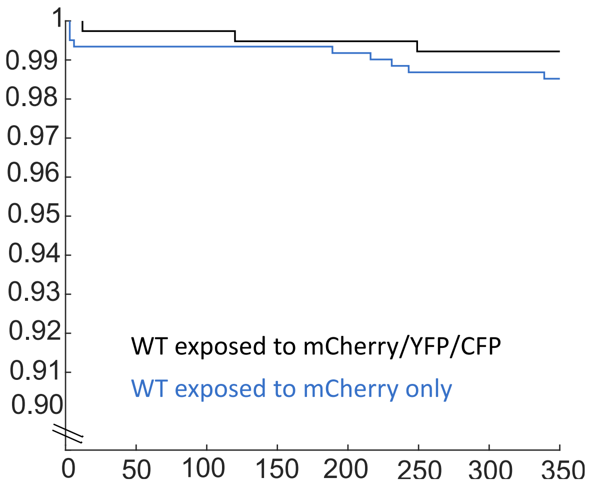

Appendix Figure S5 - YFP and CFP excitation lights do not induce toxicity

Fraction of cells surviving without treatment for WT cells exposed to CFP, YFP and mCherry light (black, 551 cells, 1 biological replicate) and WT cells exposed to mCherry light only (dark blue, 608 cells, 1 biological replicate).

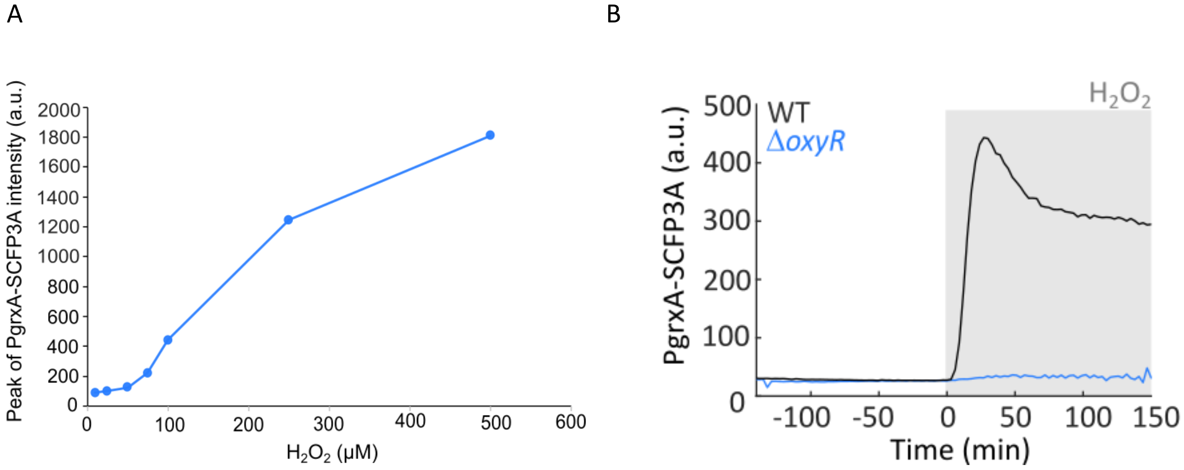

Appendix Figure S6 - PGrxA expression is dose dependent and is not induced in Δ*oxyR* mutant cells.

A Peak of PgrxA-SCFP3A reporter intensity in response to different doses of H_2O_2 (10 µM (651 cells, 1 biological replicate), 25 µM (1281 cells, 2 biological replicates), 50 µM (465 cells, 1 biological replicate), 75 µM (466 cells, 1 biological replicate), 100 µM (1848 cells, 2 biological replicates), 250 µM (331 cells, 1 biological replicate), 500 µM (764 cells, 1 biological replicate)).

B PgrxA-SCFP3A expression stays at a basal level in Δ*oxyR* cells treated with 100 µM H_2O_2 (728 cells, 3 biological replicates), compared to WT (black).

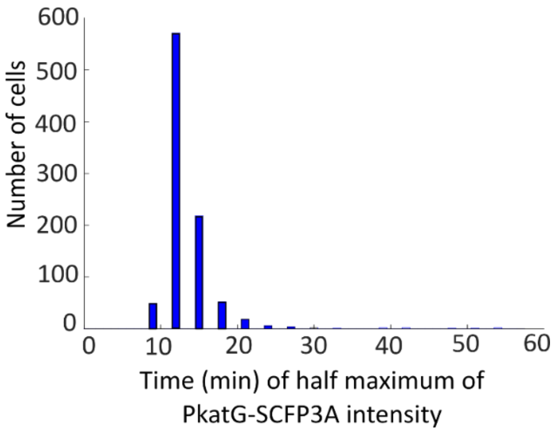

Appendix Figure S7 - KatG expression is induced shortly after the start of the treatment.

Histogram of the lag time distribution for PkatG-SCFP3A to reach half-maximal intensity after start of 100 µM H_2O_2 treatment (988 cells, 1 biological replicates).

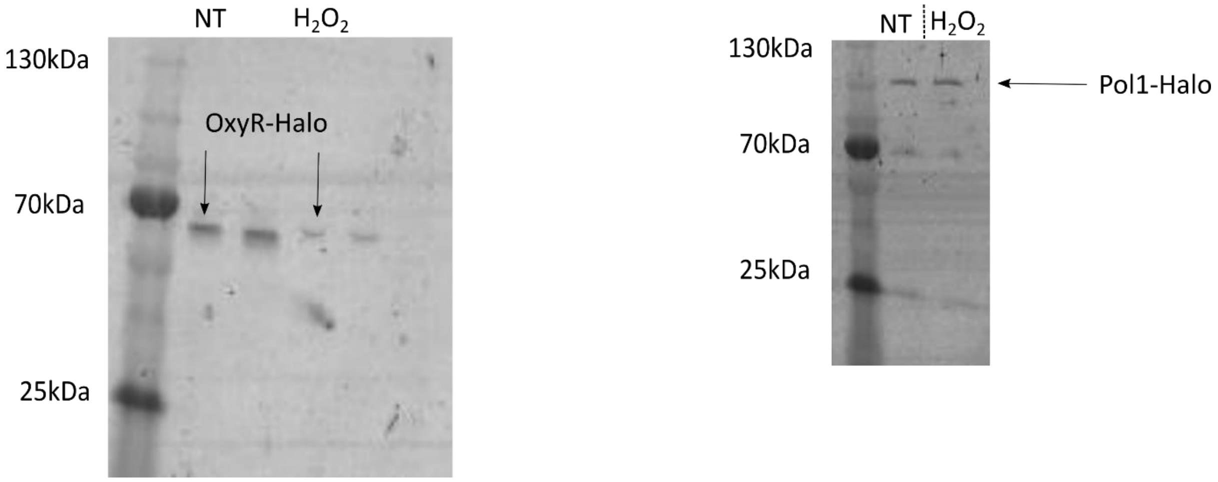

Appendix Figure S8 - **In-gel fluorescence confirms stability of OxyR-Halo and Pol1-Halo fusions.**

Strains expressing OxyR-Halo and Pol1-Halo were labelled with TMR dye as for microscopy experiments. Cells were lysed and analysed via SDS-PAGE without treatment (NT) and 30 min after treatment with 100 μM H_2O_2. For OxyR-Halo fusion, one band is detected on the gel corresponding to the expected size of OxyR (34.4 kDa) plus the HaloTag (33 kDa). For Pol1-Halo fusion, one band is detected on the gel corresponding to the expected size of Pol1 (94.08 kDa) plus the HaloTag (33 kDa). The 2 unlabelled lanes in the OxyR-Halo gel are not relevant to this work.

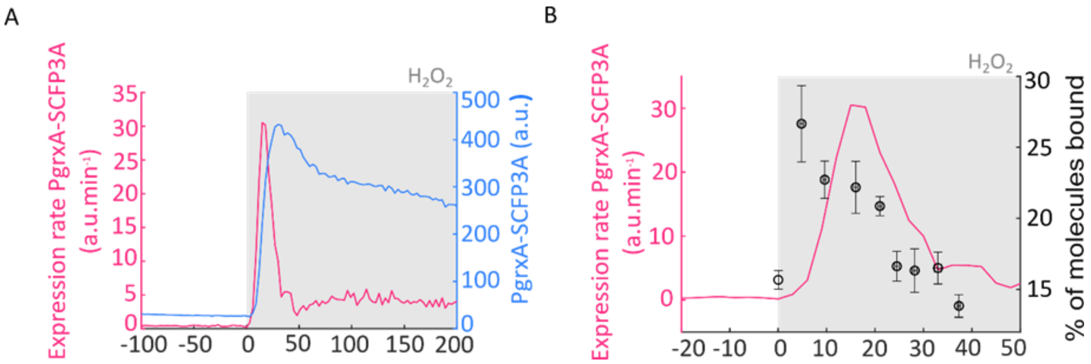

Appendix Figure S9 - Expression rate and intensity of PgrxA-mSCFP3 reporter.

A Expression rate of PgrxA-SCFP3A (pink, 878 cells**,** 1 biological replicate) and PgrxA-SCFP3A intensity (blue, 878 cells**,** 1 biological replicate) with 100 µM H_2O_2 treatment.

B Expression rate of PgrxA-SCFP3A (pink, 878 cells, 1 biological replicate) and percentage of bound OxyR-Halo molecules (black, from Fig. 3D).

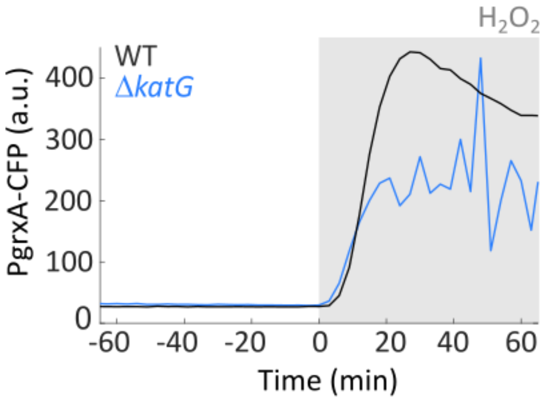

Appendix Figure S10 - PGrxA expression in Δ*katG* mutant cells.

PgrxA-SCFP3A expression in Δ*katG* cells treated with 100 μM H_2O_2 (1083 cells, 1 biological replicate), compared to WT (black).

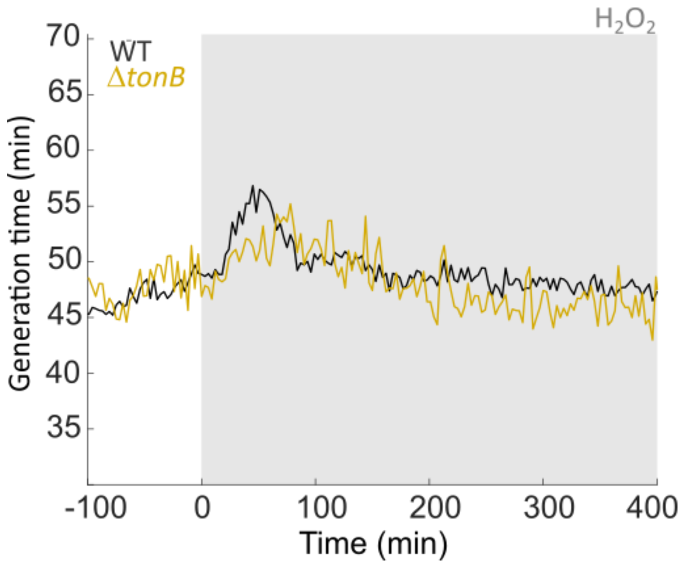

Appendix Figure S11 - Effect of iron limitation on cell cycle duration with H_2O_2 treatment.

Generation time of WT (black) and Δ*tonB* (yellow, 1753 cells, 3 biological replicates) mutant cells with 100 µM H_2O_2 treatment.

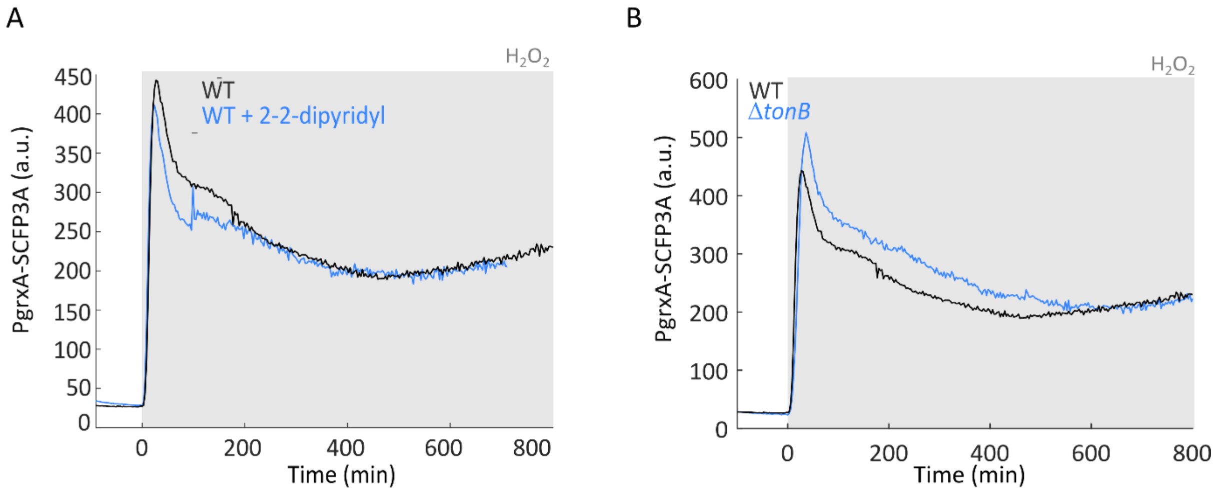

Appendix Figure S12 - Effects of perturbed iron homeostasis on OxyR response expression with 100 µM H_2O_2 treatment.

A PgrxA-SCFP3A intensity of cells in presence of DP (2270 cells, 3 experiments (biological replicates).

B PgrxA-SCFP3A intensity of Δ*tonB* mutant (573 cells, 2 biological replicates).

WT is shown in black for comparison (2355 cells, 2 biological replicates).

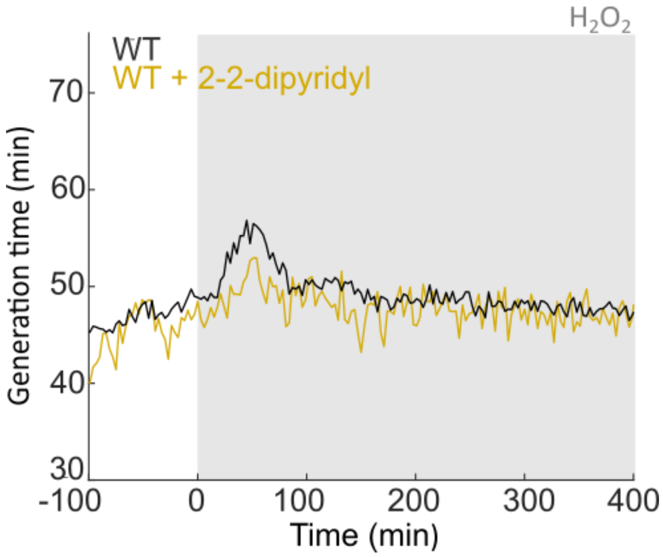

Appendix Figure S13 Effect of DP on cell cycle duration with H_2O_2 treatment.

Generation time of WT cells with 100 µM H_2O_2 treatment in medium supplemented with 50 µM 2-2-dipyridyl (yellow, 2010 cells, 3 experiments biological replicates) or without supplement (black).

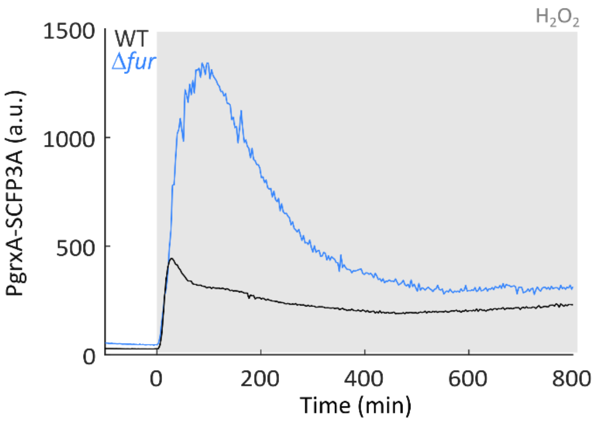

Appendix Figure S14 - Effects of *fur* deletion on OxyR response expression with 100 µM H_2O_2 treatment.

PgrxA-SCFP3A intensity of Δ*fur* mutant (520 cells, 1 biological replicate).

WT is shown in black for comparison (2355 cells, 2 biological replicates).

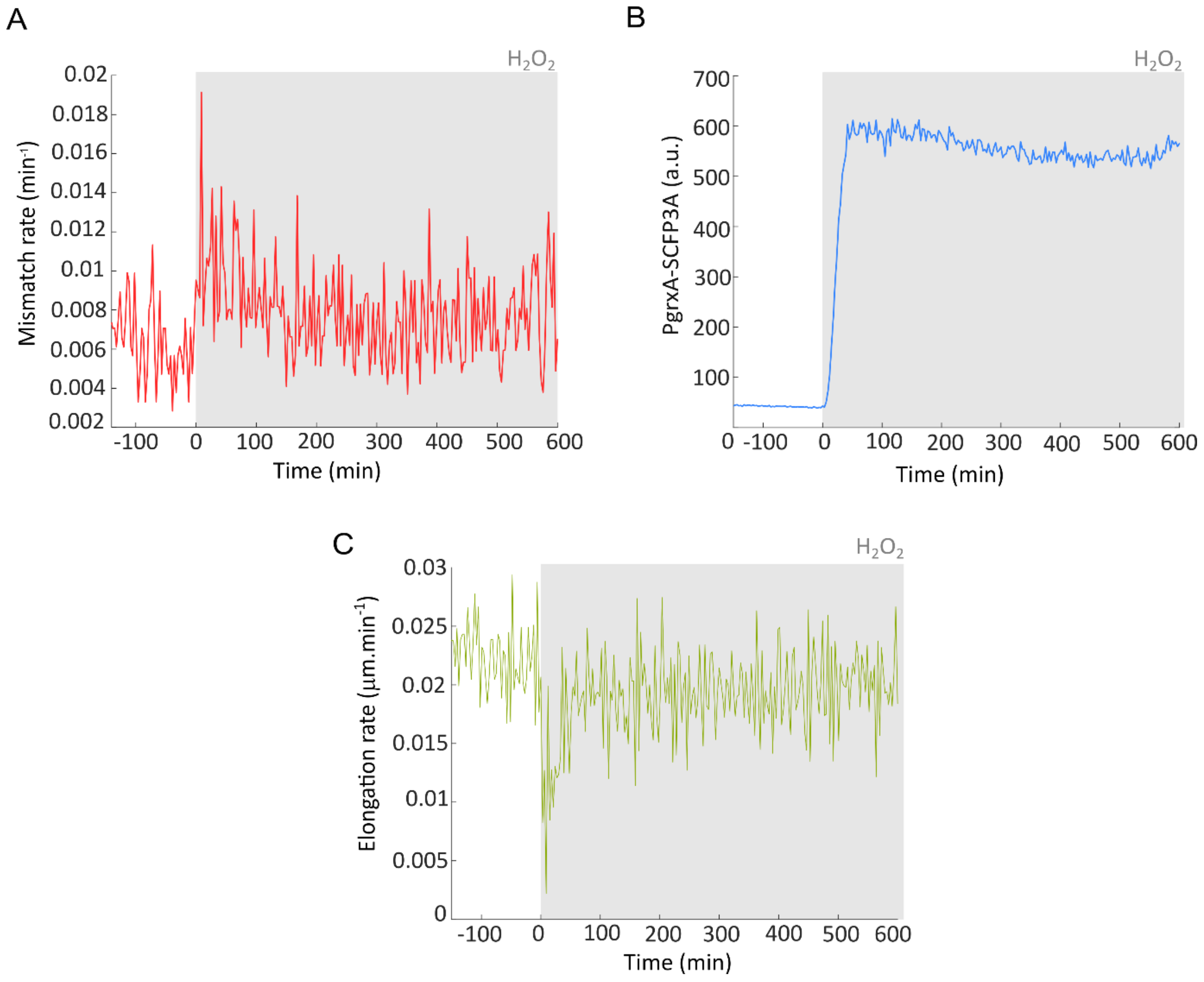

Appendix Figure S15 - Similar mismatch rate and adaptation dynamics are observed in *E. coli* strain MG1655 compared to AB1157.

A Rate of DNA mismatches per cell per minute before and during constant treatment with 100 µM H_2O_2 (710 cells, 2 biological replicates).

B PgrxA-SCFP3A expression before and during constant treatment with 100 µM H_2O_2 (710 cells, 2 biological replicates).

C Cell elongation rate before and during constant treatment with 100 µM H_2O_2 (672cells, 2 biological replicates).
