# Supplementary material for: Adaptation delay causes a burst of mutations in bacteria responding to oxidative stress
Source: EMBO Rep. 2022 Nov 17;24(1):e55640. doi: 10.15252/embr.202255640 (PMC9827559; doi:10.15252/embr.202255640)
Supplement: Supplementary file 2 — Expanded View Figures PDF [file EMBR-24-e55640-s003.pdf]

Expanded View Figures

Figure EV1. Cell elongation rate with 25 μM H_2O_2, survival after 100 min in cells treated with different doses of H_2O_2 and cell length traces showing different types of cell death with 100 μM H_2O_2 treatment.

A Cell elongation rate with 25 μM H_2O_2 (1,830 cells, three biological replicates).
B Fraction of cells surviving after 100 min of treatment with different doses of H_2O_2 (10 μM (656 cells, one biological replicate), 25 μM (1,314 cells, two biological replicates), 50 μM (476 cells, one biological replicate), 75 μM (471 cells, one biological replicate), 100 μM (6,657 cells, eight biological replicates), 250 μM (335 cells, one biological replicate), 500 μM (772 cells, one biological replicate).
C Example cell length trace showing filamentation.
D Example cell length trace showing growth arrest just before cell division.
E Example cell length trace showing growth arrest just after cell division.
F Example cell length trace showing growth arrest during the cell cycle.
G Example cell length trace showing lysis.
H Percentage of cells for each type of cell death ((6,562 cells, eight biological replicates), mean ± SEM).

© 2022 The Authors

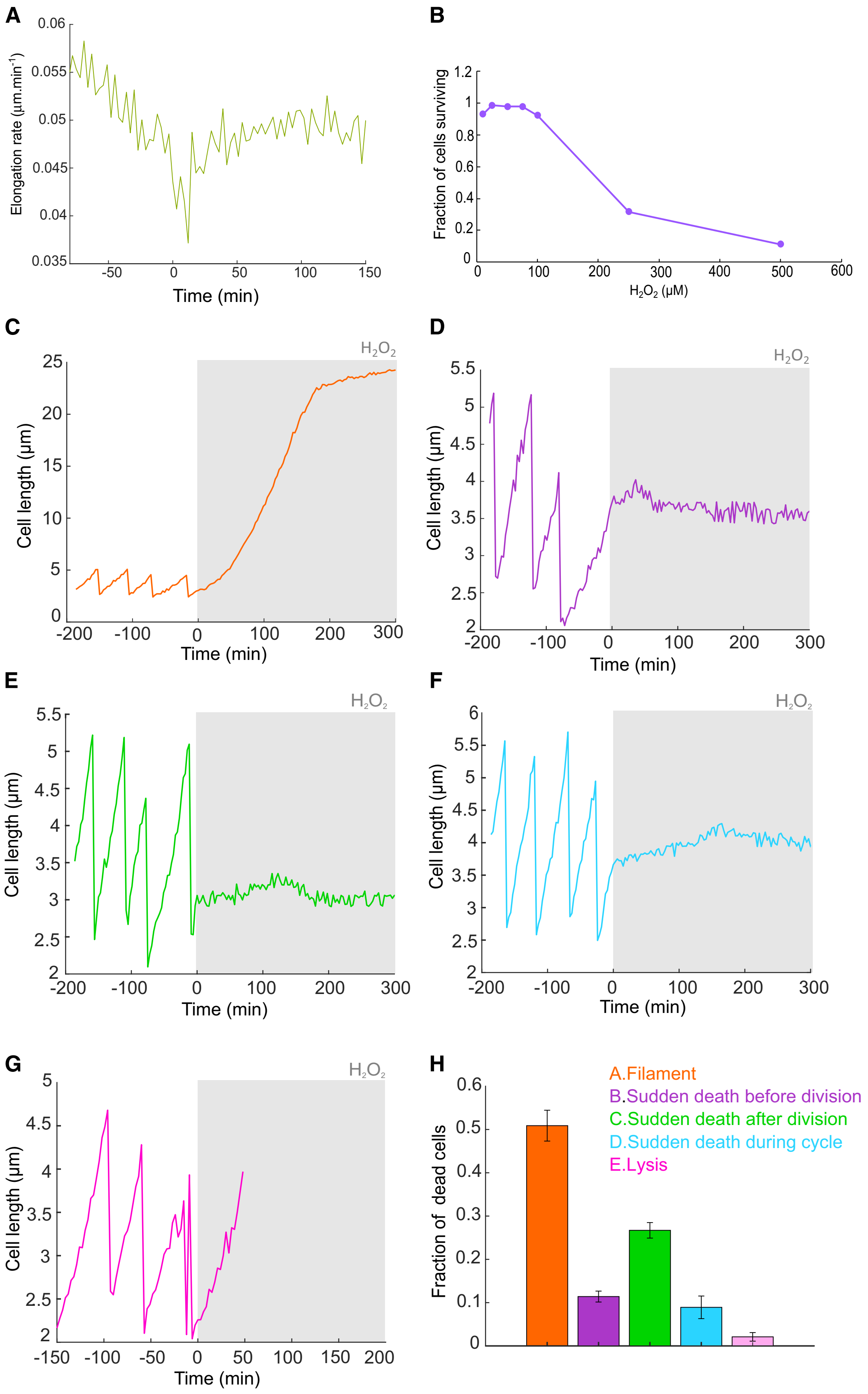

Figure EV1.

 © 2022 The Authors

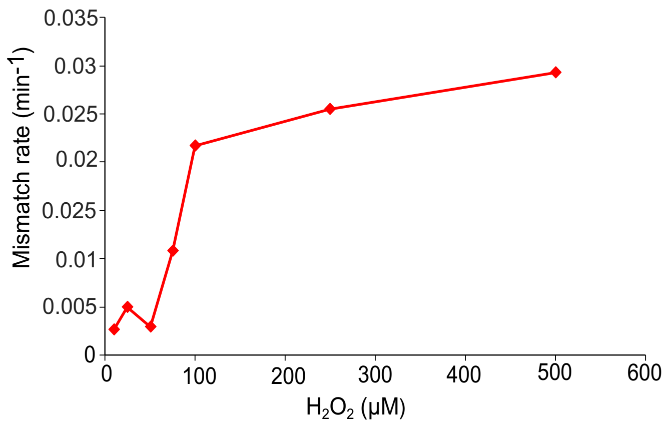

Figure EV2. Mismatch rate peak in cells treated with different doses of H_2O_2.

Rate of DNA mismatches per cell per minute at the peak of the mutagenesis burst for different doses of H_2O_2 (10 μM (651 cells, one biological replicate), 25 μM (605 cells, one biological replicate), 50 μM (one experiment, 465 cells), 75 μM (466 cells, one biological replicate), 100 μM (6,655 cells, eight biological replicates), 250 μM (331 cells, one biological replicate), 500 μM (764 cells, one biological replicate).

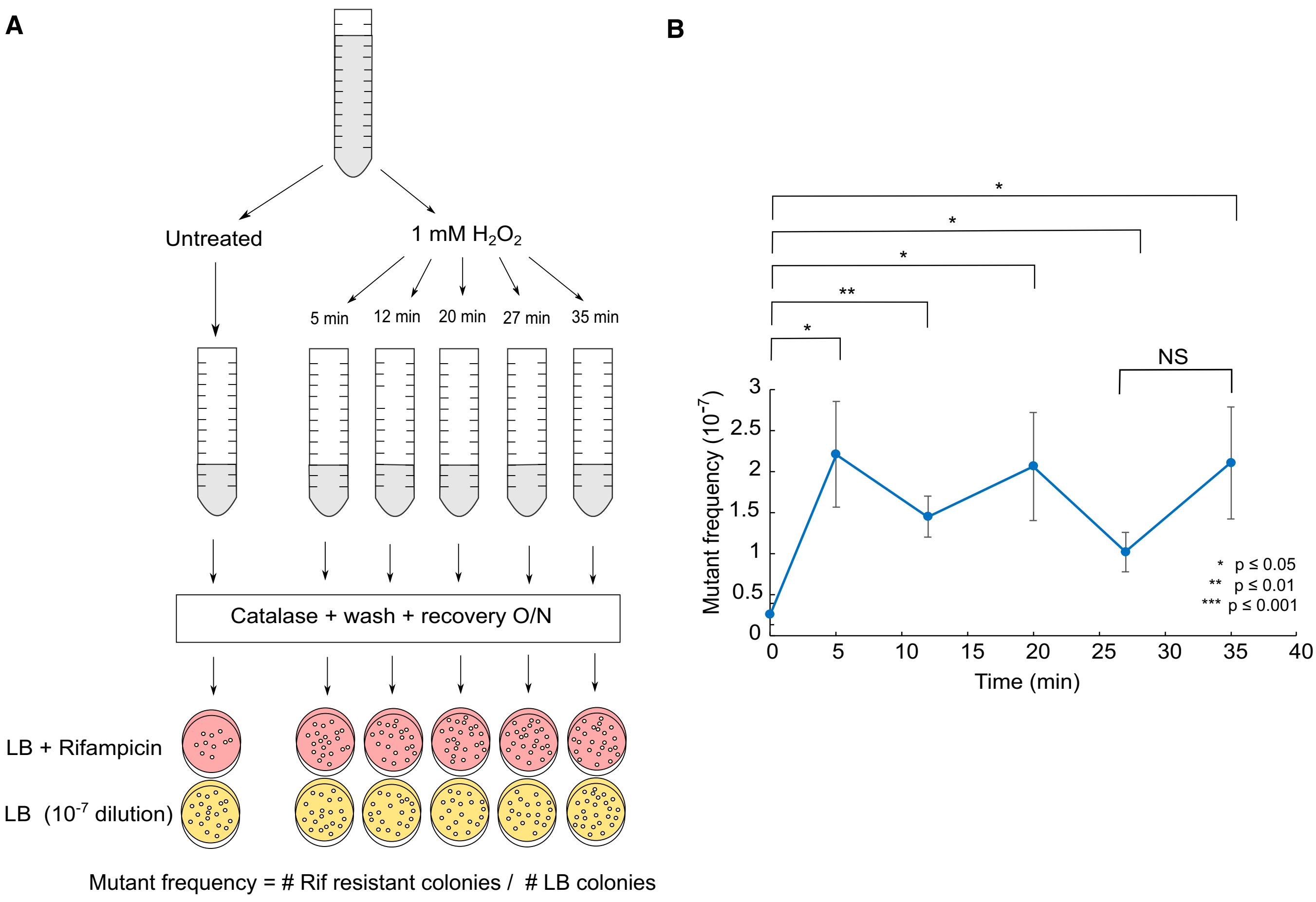

Figure EV3. Measurement of the genomic mutation frequency confirms that H_2O_2 causes a mutation burst at the start of treatment.

A Cultures were treated with 1 mM H_2O_2 and the treatment was stopped after 5, 12, 20, 27 and 32 min by adding catalase, then washed with M9 without H_2O_2 and recovered overnight before plating on LB + Rifampicin and a 10^{-7} dilution of each culture was plated on LB. Mutant frequency was quantified from the ratio of Rifampicin-resistant colonies divided by the colony count on LB plates.

B The frequency of rifampicin-resistant colonies (a reporter for mutation frequency, mean ± SEM, 6–9 experiments) increases after 5 min of 1 mM H_2O_2 treatment but does not increase further during prolonged treatment. Stars or NS (not significant) show the *P*-value of the two-sample *t*-tests.

© 2022 The Authors

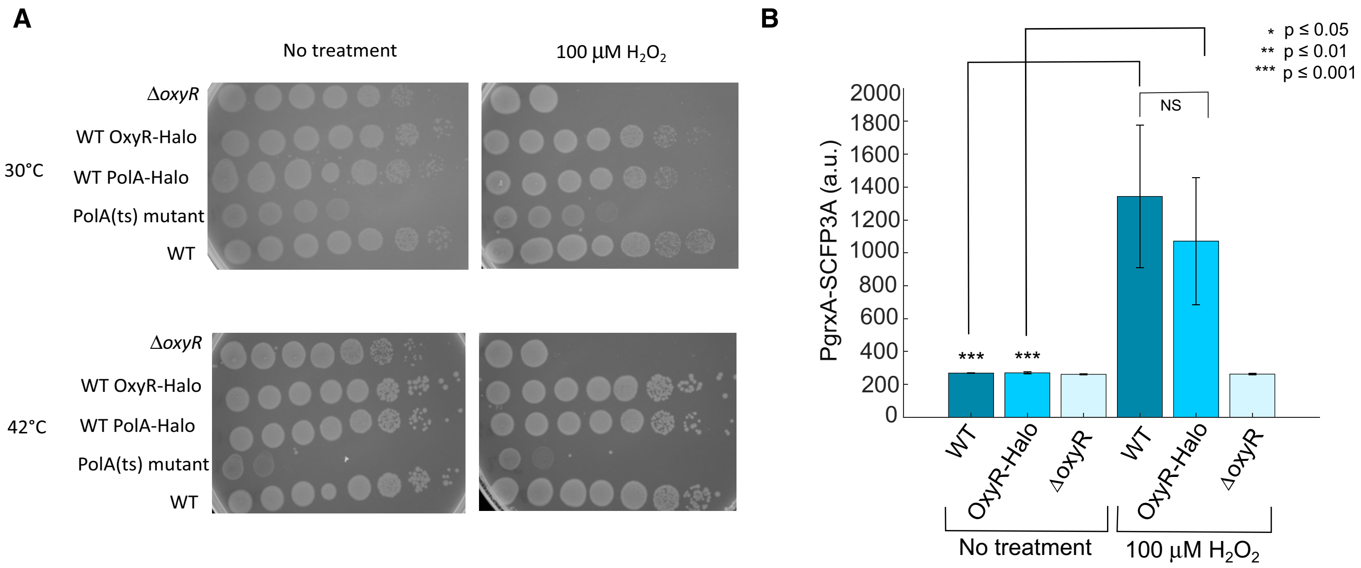

Figure EV4. Survival assay with Pol1-Halo and OxyR-Halo fusions and induction of OxyR-dependent gene expression confirms the functionality of the OxyR-Halo fusion.

A Survival assay without H_2O_2 treatment and with 100 μM H_2O_2 treatment performed at 30°C and at 42°C as PolA(ts) mutant is thermosensitive.

B Barplots of the average PgrxA-SCFP3A intensity in WT, OxyR-Halo and Δ*oxyR* mutant cells without treatment (mean ± SEM, four biological replicates, 3,748 cells for WT, 3,682 cells for WT OxyR-Halo, 2,266 cells for Δ*oxyR*) and with 100 μM H_2O_2 treatment (mean ± SEM, four biological replicates, 3,106 cells for WT, 4,336 cells, for WT OxyR-Halo, 2,816 cells for Δ*oxyR*). *P*-values of the two-sample *t*-tests are indicated or NS (not significant).

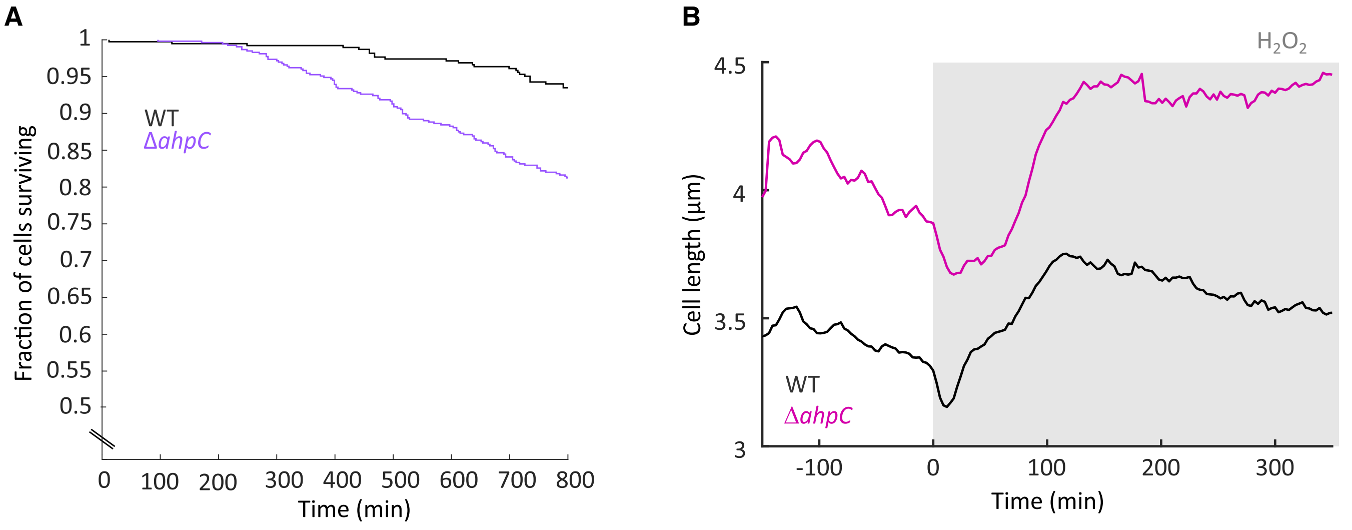

Figure EV5. Growth characteristics of Δ*ahpC* mutant cells compared with WT cells.

A Fraction of cells surviving without treatment for WT cells (black) and Δ*ahpC* mutant cells (purple, 528 cells, one biological replicate).

B The elongation rate of the Δ*ahpC* mutant is higher than for WT cells before and during 100 μM H_2O_2 treatment (Fig 4D). This can be explained by its higher cell length (B, pink, 3,360 cells, five biological replicates) compared with WT (black).

© 2022 The Authors
