# Supplementary material for: Adaptation delay causes a burst of mutations in bacteria responding to oxidative stress
Source: EMBO Rep. 2022 Nov 17;24(1):e55640. doi: 10.15252/embr.202255640 (PMC9827559; doi:10.15252/embr.202255640)
Supplement: Supplementary file 4 [file EMBR-24-e55640-s001.pdf]

EMBO reports

Adaptation delay causes a burst of mutations in bacteria responding to oxidative stress

Valentine Lagage, Victor Chen, and Stephan Uphoff
DOI: 10.15252/embr.202255640

Corresponding author: Stephan Uphoff (stephan.uphoff@bioch.ox.ac.uk)

Review Timeline:

Submission Date:	23rd Jun 22
Editorial Decision:	11th Jul 22
Revision Received:	21st Sep 22
Editorial Decision:	11th Oct 22
Revision Received:	26th Oct 22
Accepted:	28th Oct 22

Editor: Achim Breiling

Transaction Report:

(Note: With the exception of the correction of typographical or spelling errors that could be a source of ambiguity, letters and reports are not edited. Depending on transfer agreements, referee reports obtained elsewhere may or may not be included in this compilation. Referee reports are anonymous unless the Referee chooses to sign their reports.)

Dear Dr. Uphoff,

Thank you for the transfer of your research manuscript to EMBO reports. I have now received the reports from the three referees that were asked to evaluate your study, which can be found at the end of this email.

As you will see, the referees think that these findings are of interest. However, all referees have several comments, concerns, and suggestions, indicating that a major revision of the manuscript is necessary to allow publication of the study in EMBO reports. As the reports are below, and all their points need to be addressed, I will not detail them here.

Given the constructive referee comments, we would like to invite you to revise your manuscript with the understanding that all referee concerns must be addressed in the revised manuscript and in a detailed point-by-point response. Acceptance of your manuscript will depend on a positive outcome of a second round of review. It is EMBO reports policy to allow a single round of revision only and acceptance of the manuscript will therefore depend on the completeness of your responses included in the next, final version of the manuscript.

Revised manuscripts should be submitted within three months of a request for revision. Please contact me to discuss the revision (also by video chat) if you have questions or comments regarding the revision, or should you need additional time.

When submitting your revised manuscript, please also carefully review the instructions that follow below.

PLEASE NOTE THAT upon resubmission revised manuscripts are subjected to an initial quality control prior to exposition to re-review. Upon failure in the initial quality control, the manuscripts are sent back to the authors, which may lead to delays. Frequent reasons for such a failure are the lack of the data availability section (please see below) and the presence of statistics based on n=2 (the authors are then asked to present scatter plots or provide more data points).

When submitting your revised manuscript, we will require:

1) a .docx formatted version of the final manuscript text (including legends for main figures, EV figures and tables), but without the figures included. Figure legends should be compiled at the end of the manuscript text.

2) individual production quality figure files as .eps, .tif, .jpg (one file per figure), of main figures and EV figures. Please upload these as separate, individual files upon re-submission.

The Expanded View format, which will be displayed in the main HTML of the paper in a collapsible format, has replaced the Supplementary information. You can submit up to 5 images as Expanded View. Please follow the nomenclature Figure EV1, Figure EV2 etc. The figure legend for these should be included in the main manuscript document file in a section called Expanded View Figure Legends after the main Figure Legends section. Additional Supplementary material should be supplied as a single pdf file labeled Appendix. The Appendix should have page numbers and needs to include a table of content on the first page (with page numbers) and legends for all content. Please follow the nomenclature Appendix Figure Sx, Appendix Table Sx etc. throughout the text, and also label the figures and tables according to this nomenclature.

For more details, please refer to our guide to authors:
http://www.embopress.org/page/journal/14693178/authorguide#manuscriptpreparation

Please consult our guide for figure preparation:
http://wol-prod-cdn.literatumonline.com/pb-assets/embo-site/EMBOPress_Figure_Guidelines_061115-1561436025777.pdf

See also the guidelines for figure legend preparation:
https://www.embopress.org/page/journal/14693178/authorguide#figureformat

3) a complete author checklist, which you can download from our author guidelines (https://www.embopress.org/page/journal/14693178/authorguide). Please insert page numbers in the checklist to indicate where the requested information can be found in the manuscript. The completed author checklist will also be part of the RPF.

Please also follow our guidelines for the use of living organisms, and the respective reporting guidelines:
http://www.embopress.org/page/journal/14693178/authorguide#livingorganisms

4) that primary datasets produced in this study (e.g. RNA-seq, ChIP-seq, structural and array data) are deposited in an appropriate public database. If no primary datasets have been deposited, please also state this in a dedicated section (e.g. 'No primary datasets have been generated and deposited'), see below.

See also: http://embor.embopress.org/authorguide#datadeposition

Please remember to provide a reviewer password if the datasets are not yet public.

The accession numbers and database should be listed in a formal "Data Availability" section (placed after Materials & Methods) that follows the model below. This is now mandatory (like the COI statement). Please note that the Data Availability Section is restricted to new primary data that are part of this study. This section is mandatory. As indicated above, if no primary datasets have been deposited, please state this in this section

Data availability

The datasets produced in this study are available in the following databases:

- RNA-Seq data: Gene Expression Omnibus GSE46843 (https://www.ncbi.nlm.nih.gov/geo/query/acc.cgi?acc=GSE46843)
- [data type]: [name of the resource] [accession number/identifier/doi] ([URL or identifiers.org/DATABASE:ACCESSION])

*** Note - All links should resolve to a page where the data can be accessed. ***

Moreover, I have these editorial requests:

6) We strongly encourage the publication of original source data with the aim of making primary data more accessible and transparent to the reader. The source data will be published in a separate source data file online along with the accepted manuscript and will be linked to the relevant figure. If you would like to use this opportunity, please submit the source data (for example scans of entire gels or blots, data points of graphs in an excel sheet, additional images, etc.) of your key experiments together with the revised manuscript. If you want to provide source data, please include size markers for scans of entire gels, label the scans with figure and panel number, and send one PDF file per figure.

7) Our journal encourages inclusion of *data citations in the reference list* to directly cite datasets that were re-used and obtained from public databases. Data citations in the article text are distinct from normal bibliographical citations and should directly link to the database records from which the data can be accessed. In the main text, data citations are formatted as follows: "Data ref: Smith et al, 2001" or "Data ref: NCBI Sequence Read Archive PRJNA342805, 2017". In the Reference list, data citations must be labeled with "[DATASET]". A data reference must provide the database name, accession number/identifiers and a resolvable link to the landing page from which the data can be accessed at the end of the reference. Further instructions are available at: http://www.embopress.org/page/journal/14693178/authorguide#referencesformat

8) Regarding data quantification and statistics, please make sure that the number "n" for how many independent experiments were performed, their nature (biological versus technical replicates), the bars and error bars (e.g. SEM, SD) and the test used to calculate p-values is indicated in the respective figure legends (also for potential EV figures and all those in the final Appendix). Please also check that all the p-values are explained in the legend, and that these fit to those shown in the figure. Please provide statistical testing where applicable. Please avoid the phrase 'independent experiment', but clearly state if these were biological or technical replicates. See also:
http://www.embopress.org/page/journal/14693178/authorguide#statisticalanalysis

9) Please also note our reference format:
http://www.embopress.org/page/journal/14693178/authorguide#referencesformat

10) Please add scale bars of similar style and thickness to all the microscopic images (main and EV figures), using clearly visible black or white bars (depending on the background). Please place these in the lower right corner of the images themselves. Please do not write on or near the bars in the image but define the size in the respective figure legend.

11) We updated our journal's competing interests policy in January 2022 and request authors to consider both actual and perceived competing interests. Please review the policy https://www.embopress.org/competing-interests and update your competing interests if necessary. Please name this section 'Disclosure and Competing Interests Statement' and put it after the Acknowledgements section.

12) Please upload the tables 'Reagents and Tools table'. I have attached templates for that in word or excel format. Please upload the filled in table to the manuscript tracking system as a 'Reagent Table' file. The example below shows how the table will display in the published article and includes examples of the type of information that should be provided for the different categories of reagents and tools. Please list your reagents/tools using the categories provided in the template and do not add additional subheadings to the table. Reagents/tools that do not fit in any of the specific categories can be listed under "Other":
https://www.embopress.org/pb%2Dassets/embo-site/msb_177951_sample_FINAL.pdf

I look forward to seeing a revised version of your manuscript when it is ready. Please let me know if you have questions or comments regarding the revision.

Please use this link to submit your revision: Link Not Available

Yours sincerely,

Achim Breiling
Senior Editor
EMBO Reports

Referee #1:

The article "Adaptation delay causes a burst of mutations in bacteria responding to oxidative stress" is very well written. I think it contributes to understanding bacterial mutagenesis under oxidative stress and provides new methodologies. Furthermore, I believe the reported results are novel, exciting and relevant. However, like in any manuscript, there is room for improvement before presenting this article to the scientific community.

-I can intuitively understand the article's aim, but I cannot find a suitable formulation of the scientific question or the hypothesis. This is partly because the report sometimes focuses more on the method than the question.

-The authors use an interesting method using live-cell microscopy and the mother machine microfluidic device. My main concern is that the exposure of bacteria to light during the experiments could induce ROS, including continuous exposure to bacteria. The same is valid with fluorescent proteins used as reporters (see, for example, https://www.sciencedirect.com/science/article/pii/S1011134412000139). This possibility should be discussed in the manuscript as a study limitation.

-The authors used different mutants, including OxyR, ahpC and katG. AhpCF and KatG cover different ranges of H2O2 toxicity different Km. This should be discussed in the manuscript in light of the results.

- The single-molecule tracking is a good experiment. It would be great to have a more extended description of the method in the Material and Method section.

-The statement that ahpC, that in reality, the enzyme is AhpCF, is removing exogenous H2O2. Well, if H2O2 freely and quickly cross the membrane, does it matter if is endogenous or exogenous?

-The work focuses only on mutations that induce mismatches. However, Fenton chemistry also produces an important amount of DNA double-strand breaks that do not create mismatches and are repaired mainly via recombination. This should also be discussed; it may explain the quantitative discrepancy between mutagenesis level detection in the mother machine and the rifampicin assay.

-I think the article would benefit from mutation image colouring to provide a better contrast (pseudocolouring can be easily carried out with ImageJ or via microscope software).

-Line 258. However, after an initial pulse of expression at 100 min, the reporter signal returned to the basal level after ~4-5 hours of constant treatment. Therefore, DNA 260 damage levels do not remain elevated after adaptation> keep in mind here that the returning of the reporter to the basal level does not reflex the system's dynamic, only the protein's half-life. So, how stable is the fast-maturing CFP?

-Line 147. DNA mismatches become limited when cell growth is strongly inhibited by H2O2> this is true because most mutations depend on replication rate. This is also worthy to be discussed in more detail, including in some literature reports.

Referee #2:

In this paper, Lagage, Chen and Uphoff investigate the timing of phenotypic adaptation and mutagenesis of E. coli cells exposed to ROS (H2O2) via live-cell microscopy. The authors combine state of the art microfluidics and single cell microscopy to dissect the phenotypic changes and increased mutation rates induced by ROS at a great level of resolution. In general, I think this is an elegant, well-designed, and well-structured work, and I only include some minor comments below.

The only doubt/question that I have (and probably goes beyond the scope of the work), was if the authors have tried to look at how molecules that induce the production of ROS (not directly H2O2), such as antibiotics, affect the mode and tempo of ROS-induced phenotypic adaptation and mutagenesis in their experimental system? I guess the end result would be similar as the one showed for the direct addition of H2O2, but it may be interesting to check how the brief period of accelerated mutation supply could allow the cells to acquire mutations conferring resistance to the specific antibiotic used.

-In Figure4 using the same colors in panels A-D for the aphC mutants and in E-H for the pre-treated cells is a bit misleading (it seems that the authors may be using the mutants also in E-H).
-line 298, the deletion of fur gene leads to increased iron import or the fur gene leads to increased iron import? I guess it is the first one but I had to read it a few times to be sure.
-line 886, Fig. S1.
-line 955, close parenthesis.

Referee #3:

In this study, Lagage et al investigate how E. coli responds to H2O2 using single-cell microscopy. Based on a rich literature on H2O2 stress in E. coli, the authors elegantly show for the first time the adaptation to H2O2 stress in real time.

Overall, the article is well-written and the conclusions are justified, the findings from this study should be of great interest to the readership of EMBO reports. I enjoyed reading this manuscript.

There are a few minors concerns:

1. A bimodal pattern of killing by H2O2 has been reported using conventional bulk culture (doi: 10.1128/jb.166.2.519-527.1986). It would be interesting to place the results of the present study in the context of the Imlay's work. Does the observations describe here, are describing the mode-one killing? What happens if a higher concentration of H2O2 is used?

2. The Hpx mutant (katE katG ahpCF) that accumulates endogenous H2O2 shows cell elongation that has been proposed to be SOS dependent, line 249-260 the authors show that SOS induction by continuous H2O2 treatment is reduced to 100 min. How the authors interpret this difference?

3. Fig1: Align the panels to facilitate the reading of the data; Fig 1E: use an abscissa scale to see the effect at 4.5 min (line 140).

4. lines 101-103: Fig S1 it is not inhibition of cell elongation but Fraction of cells surviving

5. in the text, Fig 1D come before 1C in the text, please change.

6. the link between ROS and antibiotics is controversial, I suggest to the authors to remove this notion from the manuscript (lines 54 and 307).

7. line 165: what are the thioredoxin radical scavenging enzymes? do the authors want to talk about the trx and grx radical-scavenging activity? please clarify.

8. the results with DP are not so convincing compared to those obtained with ΔtonB. How the authors interpret this difference?

Referee #1:

The article "Adaptation delay causes a burst of mutations in bacteria responding to oxidative stress" is very well written. I think it contributes to understanding bacterial mutagenesis under oxidative stress and provides new methodologies. Furthermore, I believe the reported results are novel, exciting and relevant. However, like in any manuscript, there is room for improvement before presenting this article to the scientific community.

Thank you for the enthusiastic comments and insightful feedback.

-I can intuitively understand the article's aim, but I cannot find a suitable formulation of the scientific question or the hypothesis. This is partly because the report sometimes focuses more on the method than the question.

This is definitely something we strive to improve in our article writing. We made the following changes to clarify the central hypothesis and specific questions at several places in the manuscript:

Abstract:

"Understanding the interplay between phenotypic and genetic adaptation is a focus of evolutionary biology. In bacteria, the oxidative stress response prevents mutagenesis by reactive oxygen species (ROS). We hypothesised that the stress response dynamics could therefore affect the timing of the mutation supply that fuels genetic adaptation to oxidative stress. We uncovered that…"

Introduction (line 38):

"*Mutations can be present as pre-existing genetic variation in a population due to spontaneous mutagenesis prior to the stress exposure. However, cell stress also induces de-novo mutagenesis, which offers the potential evolutionary benefit of confining genetic changes to times when they are most likely to be beneficial. Whether stress-induced mutagenesis plays a significant role in adaptation is an open question that depends on its magnitude relative to the spontaneous mutation rate and its timing relative to the population dynamics, both of which remain uncertain. Furthermore, even well-defined treatments of bacteria with a single stress agent usually activate a complex network of stress responses involving genes that counteract mutagenesis as well as genes that actively induce mutations, making it hard to predict how the stress itself and the cellular responses together affect mutation rates. To date, experimental evidence for the actual temporal order and inter-dependence of phenotypic and genetic changes during stress adaptation is lacking because conventional methods for detecting mutations do not resolve phenotypic responses, and vice versa. In this study we asked how mutation rates change when bacteria experience stress, and how these dynamics relate to the underlying molecular mechanisms of adaptation."*

Results (line 124):

"We next asked how the timing of phenotypic adaptation relates to the rates of genetic change during oxidative stress."

Results (line 182):

"To address how the observed timing of phenotypic adaptation and mutagenesis relate to the gene expression dynamics of the OxyR response, we…"

Discussion (line 325):

"The ability of bacteria to survive these stress conditions relies on oxidative stress responses, which induce conserved scavenging enzymes that provide effective resistance to ROS such as H2O2. Nevertheless, the sublethal concentrations of H2O2 that occur frequently in the environment (Imlay, 2019) are still highly mutagenic even in wild-type bacteria with functional oxidative stress responses (Bjelland & Seeberg, 2003; Rodriguez-Rojas et al., 2020), indicating an inherent vulnerability in the

bacterial ROS defence mechanisms. Here, we set out to identify the nature of this weakness. Specifically, we asked what the chronology of H2O2-induced mutagenesis is in relation to the timing of cell killing and to the timing of the oxidative stress response in E. coli."

-The authors use an interesting method using live-cell microscopy and the mother machine microfluidic device. My main concern is that the exposure of bacteria to light during the experiments could induce ROS, including continuous exposure to bacteria. The same is valid with fluorescent proteins used as reporters (see, for example, https://www.sciencedirect.com/science/article/pii/S1011134412000139). This possibility should be discussed in the manuscript as a study limitation.

We agree with the reviewer about the potential issues of phototoxicity. To minimise this effect, our experiments have been designed such that the illumination light is switched off in between exposures. As a control, we performed time-lapse microscopy without H2O2 treatment, and found that >98% of cells survive after ~10 hours of imaging mKate2, mYPet, and SCFP3A. We have added Appendix figure S5 to show these data. We also compared survival of cells exposed to excitation light for all three fluorescence channels (mKate2, mYPet, and SCFP3A) to an otherwise identical experiment in which cells were only exposed to the mKate2 excitation light. Survival was the same in both cases, showing that the yellow and blue light imaging does not cause significant toxicity (Appendix figure S5). The reference (Waldeck et al.) mentioned by the reviewer shows that mCherry causes little photodamage (mCherry is similar to the mKate2 fluorescent protein used in our experiments). There are two other observations that show there is no significant accumulation of ROS during imaging: (i) in each experiment we can see the fluorescence intensity of oxidative stress reporter PgrxA-CFP before H2O2 treatment, and it does not increase over time; (ii) the rate of DNA mismatches was also constant over time without treatment (Appendix Fig. S2).

We have added a statement in the text (Line 182)

"To address how the observed timing of phenotypic adaptation and mutagenesis relate to the gene expression dynamics of the OxyR response, we imaged transcriptional reporters for the catalase promoter PkatG fused to the fluorescent protein SCFP3A (Balleza et al, 2018) and the glutaredoxin 1 promoter PgrxA-SCFP3A, expressed from low-copy number plasmids (Zaslaver et al, 2006). Importantly, the time-lapse fluorescence imaging did not induce reporter expression cell toxicity (Appendix figure S5), showing that our measurement conditions do not cause oxidative stress. As expected, both reporters were strongly induced during H2O2 treatment (Fig. 2G-H, Fig. EV1D), and no gene induction occurred in the ΔoxyR strain (Appendix Figure S6)."

-The authors used different mutants, including OxyR, ahpC and katG. AhpCF and KatG cover different ranges of H2O2 toxicity different Km. This should be discussed in the manuscript in light of the results.

We agree that our results provide some insight into the distinct roles of AhpCF and KatG. We have made the following changes: the manuscript to cover this discussion:

line 240:

"The mutagenesis burst was completely absent in ΔahpC cells and the mismatch rate increased gradually during constant H2O2 treatment (Fig. 4D). Hence, delayed OxyR response induction is responsible for the mutagenesis burst in WT cells. It is interesting to compare the behaviour of ΔkatG and ΔahpC strains in light of the distinct functions of the two H2O2 scavenging enzymes. Scavenging of endogenous H2O2 in untreated cells relies on the AhpCF, which has a lower enzymatic Km value than KatG (Mishra & Imlay, 2012). Indeed, whereas ΔahpC deletion leads to accumulation of endogenous H2O2 to an extent that the OxyR response becomes constitutively activated (Fig. 4A), ΔkatG deletion did not increase PgrxA reporter expression in untreated cells (Appendix Fig. S6). Although KatG is dispensable in untreated cells, the higher Km value of the enzyme allows it to scavenge H2O2 at elevated concentrations when AhpCF becomes saturated (Mishra & Imlay, 2012).

This important role of KatG is reflected in the rapid killing of ΔkatG cells during 100 µM H2O2 treatment (Fig. 2D), while the ΔahpC mutant is largely tolerant to this dose (Fig. 4C).

- The single-molecule tracking is a good experiment. It would be great to have a more extended description of the method in the Material and Method section.

We extended the description of the method in the materials and methods section. We also added a reference with a detailed protocol that we use for single-molecule tracking (Cassaro & Uphoff 2022).

(Line 565).

“Single-molecule tracking was performed using a custom-build Total Internal Reflection Fluorescence Microscope (TIRF) under oblique illumination (Banaz et al., 2019; Wegel et al, 2016) and controlled via Andor Solis and Toptica iChrome software. The microscope was constructed using ASI Modular Infinity Microscope components (MIM and RAMM, ASI). A motorized XY stage (MS2000 closed-loop with linear encoders, ASI) with a Z-piezo insert (PZ-2150, ASI) was operated by a stage and focus controller (ASI). The 100x NA 1.40 oil immersion objective (UPLSAPO, Olympus) was attached to a linear translation stage (LS50, ASI) for coarse focusing. Excitation light was provided by a multi laser engine (iChrome MLE, Toptica) with 405 nm, 488 nm, 561 nm, 640 nm lasers. Laser light exiting the optical fiber was collimated with a 150 mm visible achromatic lens (Thorlabs) and focused into the objective back-focal plane with a 200 mm visible achromatic lens (Thorlabs). Laser excitation angle could be continuously adjusted between brightfield and TIRF by moving the fibre mount and lenses perpendicular to the beam direction on a linear translation mount (Thorlabs). Excitation and emission light were separated using a multi-band dichroic mirror (zt405/488/561/640rpc, Chroma) and fluorescence emission filter (zet405/488/561/640m, Chroma) that were mounted in a filtercube (91041, Olympus). Fluorescence light was focused onto an EMCCD camera (iXon 897 Ultra, Andor) using a 300 mm achromatic tube lens module (ASI) giving a total magnification of 167x, equivalent to an image pixel size of 96 nm. For transmitted light illumination was provided by an LED light source (ASI) mounted on an Olympus IX2 condenser.

Movies of 10.000 frames with 15.48 ms time interval between frames were acquired under constant 561 nm excitation laser illumination at 0.5 kW/cm2. This induces stochastic blinking of TMR fluorophores such that only a small subset of labelled molecules is fluorescent at any time. Each field of view was exposed to 561 nm constant laser illumination at 0.5 kW/cm2 for about 30 seconds before acquisition to deactivate the majority of TMR fluorophores, providing a suitable density of fluorescent spots for single-molecule detection and tracking. The acquisition time of each movie was noted relative to the time since the cells had been placed on the agarose pad with H2O2 treatment and time points were taken approximately every 5 minutes. An image of each field of view was also acquired with transmitted light for cell segmentation. All single-molecule tracking experiments were performed at room temperature and were repeated at least three times on different days for each condition.

Single-molecule tracking data were analysed in MATLAB as previously described (Stracy et al, 2016).

Candidate positions of fluorescent molecules were identified using threshold-based spot detection in the bandpass-filtered images, followed by sub-pixel localization using a phasor algorithm (Martens et al, 2018). Localizations that appear within a spatial window of 8 pixels in consecutive frames were linked to tracks (Crocker & Grier, 1996), with a memory parameter of 1 frame (allowing for blinking or missed detection of a molecule). Cell outlines were automatically segmented from transmitted light images using a modified version of MicrobeTracker (Sliusarenko et al, 2011) combined with SuperSegger (Stylianidou et al, 2016). Any tracks with a mean position outside a segmented cell area were excluded from the analysis. The mean diffusion coefficient (D) for each track inside cells was calculated from the Mean Squared Displacement (MSD) averaged over 4 steps (5 frames): $D = MSD/(4 \cdot \Delta t)$, with Δt = 15.48 ms. Shorter tracks were discarded from the analysis and longer tracks were truncated to 4 steps. The distribution of diffusion coefficients was fitted with an analytical model of two species of molecules with diffusion coefficients D_1 and D_2 (Saxton, 1997):

$$f(x, D_1, D_2, A_1, A_2) = \frac{A_1 \left(\frac{4}{D_1}\right)^4 x^3 e^{-4x/D_1}}{6} + \frac{A_2 \left(\frac{4}{D_2}\right)^4 x^3 e^{-4x/D_2}}{6}$$

The relative abundances of the two populations A1 and A2 were constrained with A1 + A2 = 1. Fitting the combined data from all measurements and time points yielded a mean diffusion coefficient of the immobile population of molecules of D1 = 0.17 µm2/s. This value was subsequently constrained for fitting the distributions of each time point post treatment separately. The value of D2 for the mobile molecules was left unconstrained. The percentage of the immobile population A1 at each time point was used for Fig. 3D and Fig. 5D. Error bars represent the standard error of the mean (SEM) for the population percentage (on the y axis) and the time post treatment (on the x axis)."

-The statement that ahpC, that in reality, the enzyme is AhpCF, is removing exogenous H2O2. Well, if H2O2 freely and quickly cross the membrane, does it matter if is endogenous or exogenous?

We agree that it does not make a difference whether H_2O_2 is from exogenous or endogenous sources for the activity of a scavenging enzyme per se. For more clarity we have removed the second part of the sentence (Line 240).

"The mutagenesis burst was completely absent in ΔahpC cells, and the mismatch rate increased gradually during constant H2O2 treatment (Fig. 4D)."

-The work focuses only on mutations that induce mismatches. However, Fenton chemistry also produces an important amount of DNA double-strand breaks that do not create mismatches and are repaired mainly via recombination. This should also be discussed; it may explain the quantitative discrepancy between mutagenesis level detection in the mother machine and the rifampicin assay.

Indeed, oxidative DNA damage is not limited to mutagenic base lesions but also includes single-stranded and double-stranded DNA breaks. Although we focused on DNA mismatches, we also analysed the other types of damages. We used three separate assays to monitor the timing of each of these types of damages: The MutL-mYPet assay for base-pair mismatches (and short insertion-deletion loops); the Pol1-Halo assay for single-stranded breaks and DNA excision repair intermediates; the PrecA-CFP as a reporter for larger single-stranded gaps and double-stranded DNA breaks that induce the SOS response. We found that all three assays give a strong signal, consistent with the reviewer's comment that Fenton chemistry induces a large range of lesions. Interestingly, the timings of the different damage types are different: DNA mismatches are induced in a short burst for ~15 min after the start of H2O2 treatment; single-stranded breaks and excision repair intermediates are present for ~30 min; DNA gaps and breaks generate an SOS response for around ~100-300 minutes.

We modified the paragraph that describes these results to highlight the reviewer's point (line 263):

"DNA repair dynamics during oxidative stress

Oxidative DNA damage is not limited to mutagenic base lesions but also leads to single-stranded and double-stranded DNA breaks (Imlay, 2008). Our results indicate that the low rates of replication errors after an initial mutagenesis burst can be attributed to a reduction in intracellular H2O2 concentration following the upregulation of ROS scavenging enzymes. However, it is also plausible that DNA damage levels remain elevated even after adaptation but that DNA repair mechanisms efficiently remove mutagenic base lesions and strand breaks before they lead to replication errors. If so, we would expect that DNA damage levels and DNA repair activities are sustained after adaptation. To test this, we measured the expression dynamics of the SOS response which is triggered by DNA breaks (Kreuzer, 2013). H2O2 treatment induced the expression of a PrecA-SCFP3A reporter for the SOS response (Fig. 5A). However, after an initial pulse of expression at 100 min, the reporter signal

returned to the basal level after ~4-5 hours of constant treatment. Therefore, DNA damage levels do not remain elevated after adaptation.”

Regarding the mutation reporter assays: The rifampicin assay is not expected to yield data that can be quantitatively compared to the microscopy data. The key difference is that the MutL-mYPet assay detects genome-wide base-pair mismatches before they become fixed as mutations, whereas the rifampicin assay detects fixed RifR mutations in a small genomic region (the rpoB gene). Furthermore, ~99% of mismatches detected by MutL will be repaired before they become fixed as mutations. The key point is that we can compare the timing of the mutagenesis between the two assays, showing that the replication errors that lead to RifR mutations arise within 5 minutes of H2O2 treatment, similar to the duration of the MutL foci burst.

-I think the article would benefit from mutation image colouring to provide a better contrast (pseudocolouring can be easily carried out with ImageJ or via microscope software).

We have changed the pseudocolouring in figure 1D and we hope that this helps the visualisation of MutL-mYPet foci in the images.

-Line 258. However, after an initial pulse of expression at 100 min, the reporter signal returned to the basal level after ~4-5 hours of constant treatment. Therefore, DNA 260 damage levels do not remain elevated after adaptation> keep in mind here that the returning of the reporter to the basal level does not reflex the system's dynamic, only the protein's half-life. So, how stable is the fast-maturing CFP?

This is true, and we have performed an experiment to measure the decay rate of the SCFP3A reporter intensity after removal of H2O2. This is shown in Fig. S14 in a new manuscript from our group that is now available on biorxiv. (https://www.biorxiv.org/content/10.1101/2022.09.02.506373v1).

We found that the fluorescence level decays exponentially with a constant that matches the rate of cell growth and division. Hence, SCFP3A is stable, and the timing of response deactivation is dictated by the dilution of molecules due to cell division.

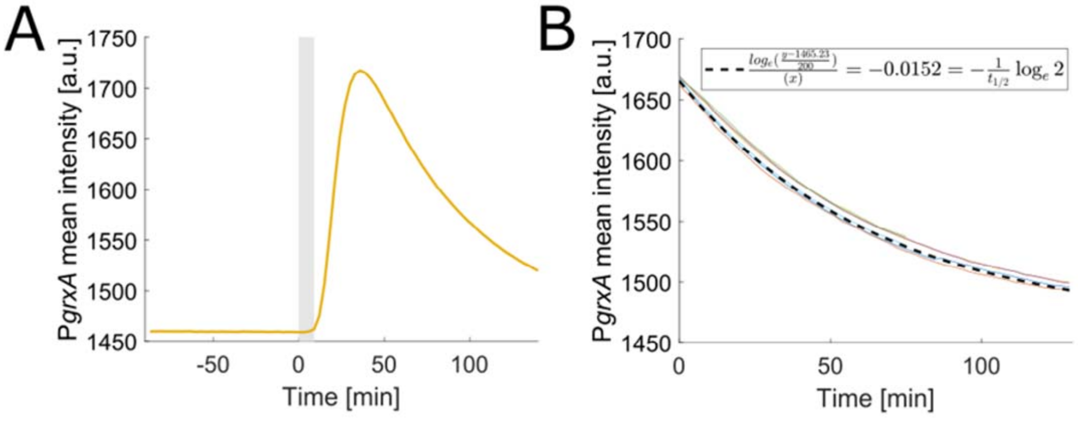

Figure caption: Exponential response decay after H2O2 removal: (A) Mean PgrxA-CFP intensity for 100 µM H2O2 treatment from 0 to 9 minutes (shaded). (B) Exponential fit to the decay of intensity after treatment removal of data shown in panel A, t1/2 = 45.6 min as expected for the generation time of ~45 min (thin lines show curve of different dilution traces and bold dashed line indicates the fit, 6 repeats).

-Line 147. DNA mismatches become limited when cell growth is strongly inhibited by H2O2> this is

true because most mutations depend on replication rate. This is also worthy to be discussed in more detail, including in some literature reports.

Indeed, a reduction in replication rate should also reduce the frequency of replication errors, and hence the rate of mismatch foci. Rapidly growing cells are generally more sensitive to DNA damage treatment. We agree with the referee, and we have added a sentence and two references in the manuscript:(Lines 151).

"The amplitude of the mutagenesis burst saturated with increasing H2O2 concentration (Fig. EV1C), indicating that the formation of mutagenic lesions or their conversion into DNA mismatches become limited when cell growth is strongly inhibited by H2O2. This is consistent with the expectation that the mismatch rate scales with the replication rate (Robert et al, 2018, Rosche & Foster, 2000)."

Referee #2:

In this paper, Lagage, Chen and Uphoff investigate the timing of phenotypic adaptation and mutagenesis of E. coli cells exposed to ROS (H2O2) via live-cell microscopy. The authors combine state of the art microfluidics and single cell microscopy to dissect the phenotypic changes and increased mutation rates induced by ROS at a great level of resolution. In general, I think this is an elegant, well-designed, and well-structured work, and I only include some minor comments below.

Thank you. We appreciate your positive comments.

The only doubt/question that I have (and probably goes beyond the scope of the work), was if the authors have tried to look at how molecules that induce the production of ROS (not directly H2O2), such as antibiotics, affect the mode and tempo of ROS-induced phenotypic adaptation and mutagenesis in their experimental system? I guess the end result would be similar as the one showed for the direct addition of H2O2, but it may be interesting to check how the brief period of accelerated mutation supply could allow the cells to acquire mutations conferring resistance to the specific antibiotic used.

This is an excellent suggestion. The reviewer will probably not be surprised that we are already working on such a project. However, the response of bacteria to antibiotics is very complicated and multi-faceted. Such a study has to be conducted with many careful controls and detailed characterisations, especially because the role of ROS in antibiotic killing is still not fully understood and controversial. An additional complexity is that contrary to H2O2 treatment, the inhibition of growth using antibiotics triggers a range of different stresses and adaptation mechanisms. We believe the single-cell imaging methods described here are ideally suited to untangle these effects. This is ongoing work in the lab and will be described in a dedicated publication. We have added a sentence in the discussion (line 362):

"With live imaging, it is possible to administer defined stress treatments such as antibiotics via microfluidics and monitor mutation supply, stress responses, cell fitness, and population dynamics simultaneously. This approach complements genetic and population-scale studies to provide direct insights into evolutionary mechanisms."

-In Figure4 using the same colors in panels A-D for the aphC mutants and in E-H for the pre-treated cells is a bit misleading (it seems that the authors may be using the mutants also in E-H).

We understand that the colours in Figure 4 can be confusing. Our rationale was to use the same colours all through the manuscript for the different mutants/conditions tested (e.g., mismatch rate is always in red, elongation rate is always in green, etc), so we wanted to keep the same colours in Figure 4 as well. Instead of changing the colours, we have added labels for panels E-H of Figure 4, "WT" and "WT pre-treated". We hope that this way the figure is clearer.

-line 298, the deletion of fur gene leads to increased iron import or the fur gene leads to increased iron import? I guess it is the first one but I had to read it a few times to be sure.

We have changed the sentence to make it clearer (Line 312).

"To probe whether mutagenesis and toxic effects were limited by the amount of intracellular iron, we increased iron import by deleting the fur gene."

-line 886, Fig. S1.

We have corrected it (now EV1, line 972)

-line 955, close parenthesis.

We have corrected it (now figure EV4, line 1018).

Referee #3:

In this study, Lagage et al investigate how E. coli responds to H2O2 using single-cell microscopy. Based on a rich literature on H2O2 stress in E. coli, the authors elegantly show for the first time the adaptation to H2O2 stress in real time.

Overall, the article is well-written and the conclusions are justified, the findings from this study should be of great interest to the readership of EMBO reports. I enjoyed reading this manuscript.

We thank the reviewer for the supportive comments and insightful questions.

There are a few minors concerns:

1. A bimodal pattern of killing by H2O2 has been reported using conventional bulk culture (doi: 10.1128/jb.166.2.519-527.1986). It would be interesting to place the results of the present study in the context of the Imlay's work. Does the observations describe here, are describing the mode-one killing? What happens if a higher concentration of H2O2 is used?

In the study by Imlay, mode-1 killing is described as occurring at low concentration of H_2O_2, to be linked to DNA damages, to be happening in metabolically active cells, and to lead to a growth lag followed by cell filamentation. Mode-2 killing, on the other hand, was described as happening with higher doses of treatment. Cells do not filament and do not restart to elongate in this case.

In our study, most of the experiments have been done with constant dose of 100 μM H_2O_2. This is different from conventional bulk culture experiments treated with a single (and usually much higher) dose of H_2O_2, so we can't directly compare the concentrations that correspond to mode-1 and mode-2 killing in Imlay's papers to our results. But since less than 10% of cells are killed in our experiments with constant 100 μM H_2O_2, this is most closely related to mode-1 killing. Our observation that DNA damages occur early after the start of treatment, and that about half of the cells that do not survive are filamenting (Fig EV2) is also consistent with mode-1 killing. We have also characterised responses at higher concentrations up to 500 mM H_2O_2 (Fig. EV1). We see that survival decreases steeply, PgrxA-CFP reporter expression increases approximately linearly, and the mismatch burst saturates (Fig. EV1). We expanded our discussion of the latter effect according to reviewer 1's comment (line 151):

"The amplitude of the mutagenesis burst saturated with increasing H2O2 concentration (Fig. EV1C), indicating that the formation of mutagenic lesions or their conversion into DNA mismatches become limited when cell growth is strongly inhibited by H2O2. This is consistent with the expectation that the mismatch rate scales with the replication rate (Rosche & Foster, 2000)."

We expanded on the concentration-dependence of cell survival (line 108):

"We observed a partial inhibition of cell elongation for H2O2 concentrations as low as 25 µM, demonstrating that physiologically-relevant H2O2 concentrations are sufficient to cause detrimental oxidative stress (Fig. EV1A). Greater than 90% of cells survived treatments up to 100 µM H2O2 but cell survival dropped steeply for higher concentrations (Fig. EV1B). For surviving cells, elongation rates recovered to pre-treatment rates after an adaptation lag period (Fig. 1C, and Appendix Figure S1)."

We also added the reference to the Imlay paper (line 120):

"However, an initial lag period before adaptation leaves the bacteria vulnerable to the toxic effects of H2O2 which have been attributed to the oxidation of DNA (Imlay & Linn 1986 and proteins (Ezraty et al, 2017) and a shutdown of protein synthesis (Zhong et al, 2015; Zhu & Dai, 2019)."

2. The Hpx mutant (katE katG ahpCF) that accumulates endogenous H2O2 shows cell elongation that has been proposed to be SOS dependent, line 249-260 the authors show that SOS induction by continuous H2O2 treatment is reduced to 100 min. How the authors interpret this difference?

We attribute this difference to the ability of wild-type cells to induce scavenging enzymes, whereas the Hpx mutant cannot. In wild-type cells, we found that the SOS response is activated only in the first 100 minutes of continuous H2O2 treatment. In these cells, the OxyR response induces the expression of the catalase and peroxidase which reduces intracellular H2O2 concentrations after an initial delay, avoiding further DNA damages and the SOS response becomes deactivated.

In a hpx mutant, the absence of katE katG ahpCF means that even low levels of endogenous H_2O_2 cannot be scavenged which leads to accumulation of DNA damage and persistent upregulation of the SOS response (which in turn causes cell filamentation).

3. Fig1: Align the panels to facilitate the reading of the data; Fig 1E: use an abscissa scale to see the effect at 4.5 min (line 140).

We have aligned the panels of Figure 1. Instead of changing the scale on Fig 1E, we have changed the abscissa scale on Fig. 2I. We thought that it is good to keep a version of the figure of the mismatch rate over a long time of treatment (showing that mismatch rate returns close to the basal rate), and one version (which is overlaid with the PgrxA intensity) which is more zoomed in on the x-axis, and so it is easier to see the pulse during the first minutes of treatment.

Just to note that the time of 4.5 min is the mean value of the peak times of different experiments, whereas Fig. 1E shows the combined data of all experiments (with a peak at 6 min). The frame interval of our measurements is 3 min. The combined data can only have a peak at a particular frame time (6 min), whereas the mean across different experiments can have a sub-frame value (4.5 min).

4. lines 101-103: Fig S1 it is not inhibition of cell elongation but Fraction of cells surviving

Thank you for spotting this error. We have added a panel with the elongation rate for 25 mM of H_2O_2 (Fig. EV1A). We have also changed the text of the manuscript, so it is coherent with the fraction of cells surviving in the Fig. EV1B (Lines 110).

"Greater than 90% of cells survived treatments up to 100 µM H2O2 but cell survival dropped steeply for higher concentrations (Fig. EV1B)."

5. in the text, Fig 1D come before 1C in the text, please change.

We have swapped the panels of Figure 1, so they now appear in the right order in the text.

6. the link between ROS and antibiotics is controversial, I suggest to the authors to remove this notion from the manuscript (lines 54 and 307).

We agree that the link between ROS and antibiotics is controversial and further work on this topic is required to resolve the debate. In this light, we think that the tools developed in this study can be very useful. Therefore, we would like to keep the mention of antibiotics, given also that reviewer 2 requested that we should discuss this topic more. But we have changed the text to acknowledge the uncertainty in this topic (Lines 59 and 323), and we avoided stating anything about whether ROS contribute to antibiotic killing or not…

"For example, sudden bursts of ROS are generated by immune cells to fight bacterial infections (Fang et al, 2016), when bacteria are exposed to radiation (Imlay, 2015) and possibly bactericidal antibiotics (Giroux et al, 2017; Hong et al, 2019; Kohanski et al, 2010b), and during competition with other bacterial species (Dong et al, 2015)."

"ROS play a key role in mediating antibacterial functions of the immune system (Fang et al., 2016), and during competition between bacterial species (Dong et al., 2015). It also has been suggested that ROS are produced when bacteria are treated with antibiotics (Kohanski, Dwyer and Collins, 2010; Giroux et al., 2017; Hong et al., 2019). "

7. line 165: what are the thioredoxin radical scavenging enzymes? do the authors want to talk about the trx and grx radical-scavenging activity? please clarify.

We agree that this sentence was unclear. Since we didn't measure Trx activity or expression, we thought it would be better to avoid confusing the reader with too many different gene names. We have simplified the sentence accordingly (now line 222):

"E. coli adapts to H2O2 by induction of ROS scavenging enzymes, which are under the control of the transcription factor OxyR (Storz et al., 1990)."

8. the results with DP are not so convincing compared to those obtained with ΔtonB. How the authors interpret this difference?

It has been shown in previous studies that DP can impact cell growth (e.g. by Rao and Kuzminov https://doi.org/10.1128/JB.00370-21). We tested several concentrations of DP and chose a moderate concentration of 50mM that does not perturb cell growth rates (Appendix Figure S10). It is likely that this concentration of DP does not completely deplete iron in the medium (and cells would attempt to upregulate iron import to maintain intracellular iron). We speculate that a deletion of the tonB gene has a stronger effect on intracellular iron levels. Although there is a difference between the two perturbations, both support the conclusion that Fe-driven Fenton chemistry is the main source of the mutagenic DNA damages.

We think that this explains the difference between DP and D*tonB* results. We have added a note in the manuscript about this point and added the reference (line 310).

"TonB deletion had a stronger effect than DP treatment because we used a moderate concentration of DP that likely reduces but not completely depletes free iron in order to maintain normal cell growth rates (Appendix Figure S10, Fig. 6A) (Rao & Kuzminov, 2022)."

1st Revision - Editorial Decision **11th Oct 2022**

Dear Dr. Uphoff,

Thank you for the submission of your revised manuscript to our editorial offices. I have received the reports from the two referees that I asked to re-evaluate your study, you will find below. As you will see, both referees now fully support the publication of your study in EMBO reports.

Before we can proceed with formal acceptance, I have these editorial requested ask you to address in a final revised version of the manuscript:

- Please provide the abstract written in present tense throughout.

- We now use CRediT to specify the contributions of each author in the journal submission system. CRediT replaces the author contribution section. Please use the free text box to provide more detailed descriptions. Thus, please remove the author contributions section from the manuscript text file. See also guide to authors: https://www.embopress.org/page/journal/14693178/authorguide#authorshipguidelines

- Please make sure that the number "n" for how many independent experiments were performed, their nature (biological versus technical replicates), the bars and error bars (e.g. SEM, SD) and the test used to calculate p-values is indicated in the respective figure legends (main, EV and Appendix figures), and that statistical testing has been done where applicable. Please avoid phrases like 'independent experiment', but clearly state if these were biological or technical replicates. Please add complete statistical testing to all diagrams (main and EV figures). Please also indicate (e.g. with n.s.) if testing was performed, but the differences are not significant. In case n=2, please show the data as separate datapoints without error bars and statistics. Presently, some diagrams seem to show only partial statistics. Moreover, could statistics be added to the diagrams shown in Figs. 5D and EV2F?

- Please name the section with the references 'References'.

- Please make sure that all figure panels are called out sequentially (alphabetically) and separately. Presently, e.g. Fig. 6J is called out after 1C, the panels of Fig. 6 are not called out sequential (alphabetical) and Fig. EV2 is called out before EV1C. Moreover, separate callouts for Figs. EV2, EV3, EV5 and Appendix Figs. S3, S10, S11, S12 are missing.

- Please remove the instructions form the reagent and tools table (e.g. "List cell lines, model organism strains"). Also empty sections (i.e. "Other") need to be deleted.

- Finally, please find attached a word file of the manuscript text (provided by our publisher) with changes we ask you to include in your final manuscript text, and some queries, we ask you to address. Please provide your final manuscript file (using the attached file as basis) with track changes, in order that we can see any modifications done.

In addition, I would need from you:
- a short, two-sentence summary of the manuscript (not more than 35 words).
- three to four short bullet points highlighting the key findings of your study (two lines each).
- a schematic summary figure (in jpeg or tiff format with the exact width of 550 pixels and a height of not more than 400 pixels) that can be used as a visual synopsis on our website.

I look forward to seeing the final revised version of your manuscript when it is ready. Please let me know if you have questions regarding the revision.

Best,

Achim Breiling
Senior Editor
EMBO Reports

Referee #1:

The authors addressed all my comments and did an extraordinary revision. From my side, I endorse the manuscript in its present form.

Referee #3:

The authors have provided comprehensive responses to the points raised in the original review and have also updated the manuscript accordingly where necessary.

Following revision I have no further comments on this manuscript.
Congratulation!

The authors addressed the minor editorial issues.

2nd Revision - Editorial Decision 28th Oct 2022

Dr. Stephan Uphoff
University of Oxford
Dept. of Biochemistry
South Parks Road
Oxford OX1 3QU
United Kingdom

Dear Dr. Uphoff,

I am very pleased to accept your manuscript for publication in the next available issue of EMBO reports. Thank you for your contribution to our journal.

At the end of this email I include important information about how to proceed. Please ensure that you take the time to read the information and complete and return the necessary forms to allow us to publish your manuscript as quickly as possible.

As part of the EMBO publication's Transparent Editorial Process, EMBO reports publishes online a Review Process File to accompany accepted manuscripts. As you are aware, this File will be published in conjunction with your paper and will include the referee reports, your point-by-point response and all pertinent correspondence relating to the manuscript.

If you do NOT want this File to be published, please inform the editorial office within 2 days, if you have not done so already, otherwise the File will be published by default [contact: emboreports@embo.org]. If you do opt out, the Review Process File link will point to the following statement: "No Review Process File is available with this article, as the authors have chosen not to make the review process public in this case." Please note that the author checklist will still be published even if you opt out of the transparent process.

Thank you again for your contribution to EMBO reports and congratulations on a successful publication. Please consider us again in the future for your most exciting work.

Yours sincerely,

Achim Breiling
Senior Editor
EMBO Reports

****

THINGS TO DO NOW:

Please note that you will be contacted by Wiley Author Services to complete licensing and payment information. The required 'Page Charges Authorization Form' is available here: https://www.embopress.org/pb-assets/embo-site/er_apc.pdf - please download and complete the form and return to embopressproduction@wiley.com

EMBO Press participates in many Publish and Read agreements that allow authors to publish Open Access with reduced/no publication charges. Check your eligibility: https://authorservices.wiley.com/author-resources/Journal-Authors/open-access/affiliation-policies-payments/index.html

You will receive proofs by e-mail approximately 2-3 weeks after all relevant files have been sent to our Production Office; you should return your corrections within 2 days of receiving the proofs.

Please inform us if there is likely to be any difficulty in reaching you at the above address at that time. Failure to meet our deadlines may result in a delay of publication, or publication without your corrections.

All further communications concerning your paper should quote reference number EMBOR-2022-55640V3 and be addressed to emboreports@wiley.com.

Should you be planning a Press Release on your article, please get in contact with emboreports@wiley.com as early as possible, in order to coordinate publication and release dates.

EMBO Press Author Checklist

Corresponding Author Name: Stephan Uphoff	
Journal Submitted to: EMBO reports	
Manuscript Number: EMBOR-2022-55640-T	

USEFUL LINKS FOR COMPLETING THIS FORM
The EMBO Journal - Author Guidelines
EMBO Reports - Author Guidelines
Molecular Systems Biology - Author Guidelines
EMBO Molecular Medicine - Author Guidelines

Reporting Checklist for Life Science Articles (updated January 2022)
This checklist is adapted from Materials Design Analysis Reporting (MDAR) Checklist for Authors. MDAR establishes a minimum set of requirements in transparent reporting in the life sciences (see Statement of Task: 10.31222/osf.io/9sm4x). Please follow the journal's guidelines in preparing your manuscript.
Please note that a copy of this checklist will be published alongside your article.

Abridged guidelines for figures
1. Data
The data shown in figures should satisfy the following conditions:
- the data were obtained and processed according to the field's best practice and are presented to reflect the results of the experiments in an accurate and unbiased manner.
- ideally, figure panels should include only measurements that are directly comparable to each other and obtained with the same assay.
- plots include clearly labeled error bars for independent experiments and sample sizes. Unless justified, error bars should not be shown for technical replicates.
- if n<5, the individual data points from each experiment should be plotted. Any statistical test employed should be justified.
- Source Data should be included to report the data underlying figures according to the guidelines set out in the authorship guidelines on Data Presentation.

2. Captions
Each figure caption should contain the following information, for each panel where they are relevant:
- a specification of the experimental system investigated (eg cell line, species name).
- the assay(s) and method(s) used to carry out the reported observations and measurements.
- an explicit mention of the biological and chemical entity(ies) that are being measured.
- an explicit mention of the biological and chemical entity(ies) that are altered/varied/perturbed in a controlled manner.
- the exact sample size (n) for each experimental group/condition, given as a number, not a range;
- a description of the sample collection allowing the reader to understand whether the samples represent technical or biological replicates (including how many animals, litters, cultures, etc.).
- a statement of how many times the experiment shown was independently replicated in the laboratory.
- definitions of statistical methods and measures:
  - common tests, such as t-test (please specify whether paired vs. unpaired), simple χ2 tests, Wilcoxon and Mann-Whitney tests, can be unambiguously identified by name only, but more complex techniques should be described in the methods section;
  - are tests one-sided or two-sided?
  - are there adjustments for multiple comparisons?
  - exact statistical test results, e.g., P values = x but not P values < x;
  - definition of 'center values' as median or average;
  - definition of error bars as s.d. or s.e.m.

Please complete ALL of the questions below.
Select "Not Applicable" only when the requested information is not relevant for your study.

Materials

Newly Created Materials	Information included in the manuscript?	In which section is the information available? (Reagents and Tools Table, Materials and Methods, Figures, Data Availability Section)
New materials and reagents need to be available; do any restrictions apply?	Yes	Reagents and Tools Table and Materials and Methods (p9)

Antibodies	Information included in the manuscript?	In which section is the information available? (Reagents and Tools Table, Materials and Methods, Figures, Data Availability Section)
For **antibodies** provide the following information:<br>- Commercial antibodies: RRID (if possible) or supplier name, catalogue number and or/clone number<br>- Non-commercial: RRID or citation	Not Applicable	

DNA and RNA sequences	Information included in the manuscript?	In which section is the information available? (Reagents and Tools Table, Materials and Methods, Figures, Data Availability Section)
**Short novel DNA or RNA including primers, probes**: provide the sequences.	Yes	Reagents and Tools Table, Materials and Methods (p9-10)

Cell materials	Information included in the manuscript?	In which section is the information available? (Reagents and Tools Table, Materials and Methods, Figures, Data Availability Section)
**Cell lines:** Provide species information, strain. Provide accession number in repository **OR** supplier name, catalog number, clone number, and/**OR** RRID.	Not Applicable	
**Primary cultures:** Provide species, strain, sex of origin, genetic modification status.	Not Applicable	
Report if the cell lines were recently **authenticated** (e.g., by STR profiling) and tested for mycoplasma contamination.	Not Applicable	

Experimental animals	Information included in the manuscript?	In which section is the information available? (Reagents and Tools Table, Materials and Methods, Figures, Data Availability Section)
**Laboratory animals or Model organisms:** Provide species, strain, sex, age, genetic modification status. Provide accession number in repository **OR** supplier name, catalog number, clone number, **OR** RRID.	Not Applicable	
**Animal observed in or captured from the field:** Provide species, sex, and age where possible.	Not Applicable	
Please detail **housing and husbandry conditions**.	Not Applicable	

Plants and microbes	Information included in the manuscript?	In which section is the information available? (Reagents and Tools Table, Materials and Methods, Figures, Data Availability Section)
**Plants:** provide species and strain, ecotype and cultivar where relevant, unique accession number if available, and source (including location for collected wild specimens).	Not Applicable	
**Microbes:** provide species and strain, unique accession number if available, and source.	Yes	Escherichia coli AB1157 from colleague (David Sherratt). Reagents and Tools Table, Materials and Methods (p9)

Human research participants	Information included in the manuscript?	In which section is the information available? (Reagents and Tools Table, Materials and Methods, Figures, Data Availability Section)
If collected and within the bounds of privacy constraints report on age, sex and gender or ethnicity for all study participants.	Not Applicable	

Core facilities	Information included in the manuscript?	In which section is the information available? (Reagents and Tools Table, Materials and Methods, Figures, Data Availability Section)
If your work benefited from core facilities, was their service mentioned in the acknowledgments section?	Yes	Acknowledgement section (p14)

Design

Study protocol	Information included in the manuscript?	In which section is the information available? (Reagents and Tools Table, Materials and Methods, Figures, Data Availability Section)
If study protocol has been **pre-registered, provide DOI in the manuscript**. For clinical trials, provide the trial registration number **OR** cite DOI.	Not Applicable	
Report the **clinical trial registration number** (at ClinicalTrials.gov or equivalent), where applicable.	Not Applicable	

Laboratory protocol	Information included in the manuscript?	In which section is the information available? (Reagents and Tools Table, Materials and Methods, Figures, Data Availability Section)
Provide DOI OR other citation details if **external detailed step-by-step protocols** are available.	Yes	Cassaro, C. J. & Uphoff, S. Super-Resolution Microscopy and Tracking of DNA-Binding Proteins in Bacterial Cells. Methods Mol Biol 2476, 191–208 (2022).

Experimental study design and statistics	Information included in the manuscript?	In which section is the information available? (Reagents and Tools Table, Materials and Methods, Figures, Data Availability Section)
Include a statement about **sample size** estimate even if no statistical methods were used.	Yes	Figure legends (p18-21), Appendix figure legends, Materials and Methods (p12-14)
Were any steps taken to minimize the effects of subjective bias when allocating animals/samples to treatment (e.g. **randomization procedure**)? If yes, have they been described?	Not Applicable	
Include a statement about **blinding** even if no blinding was done.	Not Applicable	
Describe **inclusion/exclusion criteria** if samples or animals were excluded from the analysis. Were the criteria pre-established?<br><br>If sample or data points were omitted from analysis, report if this was due to attrition or intentional exclusion and provide justification.	Yes	Data from experiments with technical failure (e.g. microscope malfunction) were excluded.
For every figure, are **statistical tests** justified as appropriate? Do the data meet the assumptions of the tests (e.g., normal distribution)? Describe any methods used to assess it. Is there an estimate of variation within each group of data? Is the variance similar between the groups that are being statistically compared?	Yes	Figure legends (p18-21), Appendix figure legends, Materials and Methods (p12-14)

Sample definition and in-laboratory replication	Information included in the manuscript?	In which section is the information available? (Reagents and Tools Table, Materials and Methods, Figures, Data Availability Section)
In the figure legends: state number of times the experiment was **replicated** in laboratory.	Yes	Figure legends (p18-21), Appendix figure legends
In the figure legends: define whether data describe **technical or biological replicates**.	Yes	Figure legends (p18-21), Appendix figure legends

Ethics

Ethics	Information included in the manuscript?	In which section is the information available? (Reagents and Tools Table, Materials and Methods, Figures, Data Availability Section)
Studies involving **human participants**: State details of **authority granting ethics approval** (IRB or equivalent committee(s), provide reference number for approval.	Not Applicable	
Studies involving **human participants**: Include a statement confirming that **informed consent** was obtained from all subjects and that the experiments conformed to the principles set out in the WMA Declaration of Helsinki and the Department of Health and Human Services Belmont Report.	Not Applicable	
Studies involving **human participants:** For publication of **patient photos**, include a statement confirming that consent to publish was obtained.	Not Applicable	
Studies involving experimental **animals**: State details of **authority granting ethics approva**l (IRB or equivalent committee(s), provide reference number for approval. Include a statement of compliance with ethical regulations.	Not Applicable	
Studies involving **specimen and field samples:** State if relevant **permits** obtained, provide details of authority approving study; if none were required, explain why.	Not Applicable	

Dual Use Research of Concern (DURC)	Information included in the manuscript?	In which section is the information available? (Reagents and Tools Table, Materials and Methods, Figures, Data Availability Section)
Could your study fall under dual use research restrictions? Please check biosecurity documents and list of **select agents and toxins** (CDC): https://www.selectagents.gov/sat/list.htm	Not Applicable	
If you used a select agent, is the security level of the lab appropriate and reported in the manuscript?	Not Applicable	
If a study is subject to dual use research of concern regulations, is the name of the **authority granting approval and reference number** for the regulatory approval provided in the manuscript?	Not Applicable	

Reporting

The MDAR framework recommends adoption of discipline-specific guidelines, established and endorsed through community initiatives. Journals have their own policy about requiring specific guidelines and recommendations to complement MDAR.

Adherence to community standards	Information included in the manuscript?	In which section is the information available? (Reagents and Tools Table, Materials and Methods, Figures, Data Availability Section)
State if relevant guidelines or checklists (e.g., **ICMJE, MIBBI, ARRIVE, PRISMA**) have been followed or provided.	Not Applicable	
For **tumor marker prognostic studies,** we recommend that you follow the **REMARK** reporting guidelines (see link list at top right). See author guidelines, under 'Reporting Guidelines'. Please confirm you have followed these guidelines.	Not Applicable	
For **phase II and III randomized controlled trials**, please refer to the **CONSORT** flow diagram (see link list at top right) and submit the CONSORT checklist (see link list at top right) with your submission. See author guidelines, under 'Reporting Guidelines'. Please confirm you have submitted this list.	Not Applicable	

Data Availability

Data availability	Information included in the manuscript?	In which section is the information available? (Reagents and Tools Table, Materials and Methods, Figures, Data Availability Section)
Have **primary datasets** been deposited according to the journal's guidelines (see 'Data Deposition' section) and the respective accession numbers provided in the Data Availability Section?	Yes	Data availability section (p14)
Were **human clinical and genomic datasets** deposited in a public access-controlled repository in accordance to ethical obligations to the patients and to the applicable consent agreement?	Not Applicable	
Are **computational models** that are central and integral to a study available without restrictions in a machine-readable form? Were the relevant accession numbers or links provided?	Not Applicable	
If publicly available data were reused, provide the respective **data citations in the reference list**.	Not Applicable	
